# Bayesian Cost-Effectiveness Analysis Using Individual-Level Data is Sensitive to the Choice of Uniform Priors on the Standard Deviations for Costs in Log-Normal Models

**DOI:** 10.1007/s40273-025-01511-1

**Published:** 2025-08-12

**Authors:** Xiaoxiao Ling, Andrea Gabrio, Gianluca Baio

**Affiliations:** 1 Nuffield Department of Primary Care Health Sciences, University of Oxford, Radcliffe Observatory Quarter, Woodstock Road, Oxford, OX2 6GG UK; 2 Department of Methodology and Statistics, Faculty of Health Medicine and Life Science, Maastricht University, Peter Debyeplein 1, 6229 HA Maastricht, Limburg, The Netherlands; 3 Department of Statistical Science, University College London, 1-19 Torrington Place, London, WC1E 6BT UK

## Abstract

**Background:**

Bayesian cost-effectiveness analysis (CEA) requires the specification of prior distributions for all parameters to be empirically estimated via Bayes’ rule. When costs are modelled via Log-Normal distributions, Uniform prior distributions are commonly applied on the logarithm-scale standard deviations for costs due to the ease of implementation. However, the consequences of placing wide Uniform priors on standard deviations of log costs for the interpretation of original-scale CEA results remain unclear. The purpose of our study is to explore the impact of using Uniform priors for the standard deviations of cost data on CEA conclusions when costs are assumed to be log-normally distributed.

**Methods:**

The analysis has been performed using individual-level cost-utility data from a randomised controlled trial. Costs are initially jointly modelled with quality-adjusted life years (QALYs) using Log-Normal and Beta distributions, respectively. Uniform prior distributions with different upper bounds are applied to log-scale standard deviations in the cost Log-Normal model. We compare the performance of Uniform priors under the Log-Normal distribution with other distributional assumptions for costs. A simulation study has then been conducted to explore the impact of these models and prior choices on cost estimates in CEAs.

**Results:**

Results show that the choice of Uniform priors on standard deviations of log costs in a Log-Normal model can substantially induce large fluctuations in cost estimates, and thus potentially affect the final estimates of the intervention being cost-effective compared with other distributional assumptions. This is potentially driven by the occurrence of zero values in cost data.

**Conclusion:**

Bayesian CEAs may be sensitive to the choice of upper bounds of the Uniform priors for the standard deviations of log costs in Log-Normal models, particularly when data contain zero values. Our results suggest that caution should be taken when Uniform distributions with large upper bounds are used.

**Supplementary Information:**

The online version contains supplementary material available at 10.1007/s40273-025-01511-1.

## Key Points for Decision Makers

**Table:** 

Bayesian cost-effectiveness analyses are sensitive to the choice of upper bounds of the Uniform prior distributions on log cost standard deviations when costs data are assumed to be log-normally distributed.
Caution should be given to the potential consequences of applying Uniform distributions on cost standard deviations as minimally informative priors based on both distributional model assumptions and actual characteristics of the data.
It is more conservative to choose models that are more robust to the upper bounds of Uniform prior distributions and it is also necessary to consider alternative priors to assess the robustness of posterior results.

## Introduction

Bayesian analysis has become increasingly popular in cost-effectiveness analysis (CEA) over the years [1–4]. One of the reasons behind its popularity stems from its ability to incorporate external evidence or elicited expert opinions into the analysis in a principled way, through prior distributions. However, in cases where genuine information is lacking, ‘minimally informative’ prior distributions are often used, at least as a starting point [5, 6].

While common in Bayesian CEAs [7], the implementation of models based on scarce contextual information in real applications can be challenging. Given that CEA data are usually skewed, simple assumptions such as a Normal distribution to describe sampling variability are less likely to hold. Alternative choices (e.g. modelling sampling variability using a Gamma distribution to cater for skewness and positivity of the individual costs) are often specified using classical parameters whose ‘physical’ interpretations, while meaningful, may be less immediately intuitive to health economists. For instance, unlike the Normal distribution which is naturally parameterised by a population mean and standard deviation, a Gamma distribution is usually indexed by a *rate* and a *shape* parameter (rather than its mean and standard deviation), which are difficult to understand in a health economics context and complicate the natural interpretation and elicitation of prior beliefs [8].

The Uniform distribution on a suitable large and positive range is often considered as the default choice for a minimally informative prior concerning the standard deviation of costs [9–11]. However, slight differences exist in the specification of the Uniform prior under different distributional assumptions in the analysis of cost data. Uniform priors tend to be placed on the original scale of standard deviations or their functions when costs are assumed to follow a Normal or Gamma distribution [8–10, 12], while it is straightforward to directly assign priors on standard deviations of log costs when costs are modelled through a Log-Normal distribution [13].

In the general Bayesian statistical literature, it has been already recognised that placing vague Uniform priors on log-scale standard deviations in Log-Normal models is often a sensitive choice, which exerts a potentially notable influence on posterior results [14, 15]. Alternative options of minimally informative priors for variance or standard deviation have been suggested in the wider statistical literature. For example, Gelman recommended the half-t family of prior distributions on the original-scale standard deviation [16], but the article primarily focused on hierarchical Normal models, which may not always align with CEA data. Under the choice of a Log-Normal distribution, Zellner proposed the use of diffuse priors [17], although the method encounters the difficulty of summarising the posterior distribution of the mean on its original scale. Fabrizi and Trivisano proposed a generalised inverse Gaussian prior on the log-scale variance of the Log-Normal distribution, allowing for finite posterior mean estimates [18]. Yet, the application of these priors from statistical literature to CEAs poses challenges in interpretation since they are less intuitive compared with a Uniform prior distribution.

The objective of this study is to investigate how setting Uniform priors for the standard deviations of a cost Log-Normal distribution may affect the overall CEAs. Our focus is different from identifying the ‘optimal’ prior from a statistical viewpoint but is more specialised in the context of health economic evaluations. So far, there has been little discussion regarding the choice of prior distributions in CEAs. It remains poorly understood to what extent the application of Uniform distributions as minimally informative priors for standard deviations of log costs in Log-Normal models can function to easily convey specific beliefs about cost standard deviations on their natural scale.

The study will explore the impact of using Uniform prior distributions for the log-scale standard deviations of cost data on cost-effectiveness results when costs are assumed to be log-normally distributed using a real case study. The Log-Normal model will be compared with models with other distributional assumptions in terms of their CEA results. Based on these, recommendations will be formulated regarding the use of Uniform prior distributions for cost standard deviations in Bayesian CEAs.

## Materials and Methods

### Case Study

The ORBIT trial is a randomised controlled trial that compares the cost-effectiveness of the intervention, online-delivered therapist-supported exposure and response prevention, with the control, online education, among children with Tourette syndrome or chronic tics disorder from a health care perspective over 6 months [19]. A total of 224 patients had been randomly assigned to the two treatment arms: 112 were randomised to the intervention and 112 to the control. Cost and utility data were collected at baseline, 3 months and 6 months. The total costs and quality-adjusted life years (QALYs) [20] were estimated at 6 months using the cost and utility data at each time point.

Complete cases, defined as patients with fully observed health care costs and utility scores at the three data collection time points, were used to streamline the analysis (71 in the control and 62 in the intervention). The decision was made because we concentrated on providing a comprehensive insight into how the choice of Uniform priors with various upper bounds can influence cost-effectiveness conclusions and considered that a complete case analysis was adequate to serve this purpose.

### Statistical Models

Analyses were performed within a Bayesian framework using a joint modelling approach [21]. The joint distribution of costs ($c$) and QALYs ($e$), denoted as $p\left(c,e\right)$, was parameterised by the product of a conditional distribution of costs given QALYs, $p\left(c\mid e\right)$, and a marginal distribution of QALYs, $p\left(e\right)$, to capture the correlation between the outcomes. Apart from the Log-Normal distribution, Normal and Gamma distributions were considered for cost data to illustrate the extent to which different distributional assumptions can influence the impact of Uniform priors for cost standard deviations on cost-effectiveness results. Normal and Beta distributions were applied to QALYs; the former was chosen following the original health economic analysis of the trial, while the latter was used to reflect the potential skewness of QALYs.

The following notation was used: let ${c}_{it}$ and ${e}_{it}$ denote total costs and QALYs for patient $i$ in each treatment arm $t$ respectively. The treatment indicator $t$ was dropped to simplify model specification.

#### Normal Model

The first model, assuming that both costs and QALYs are normally distributed, was undertaken to mimic the assumptions of routine CEAs. It can be thought of as a Bayesian equivalent to *seemingly unrelated regression* (SUR) [22] but re-expressed by using the aforementioned product formulation of the conditional distribution of costs given QALYs and the distribution of QALYs.

We modelled costs conditional on QALYs as:

$$\begin{array}{ll}{c}_{i}\mid {e}_{i} \sim {\text{Normal}}\left({\mu }_{ci},{\sigma }_{c}^{2}\right)\\ {\mu }_{ci}={\alpha }_{0}+{\alpha }_{1}\left({c}_{0i}-{\overline{c} }_{0}\right)+{\alpha }_{2}\left({e}_{i}-\overline{e }\right)+{\alpha }_{3}{s}_{i}\end{array}$$

where ${\mu }_{ci}$ and ${\sigma }_{c}$ are the (individual-level) mean and the (population-level) standard deviation of the costs. Covariates were selected based on the original health economics analysis: $\left( {c_{0i} - \overline{c}_{0} } \right)$ is the centred baseline costs, $\left( {e_{i} - \overline{e}} \right)$ is the centered QALYs while ${s}_{i}$ is the site at which individual $i$ has been treated. ${\alpha }_{0}$ is the intercept, ${\alpha }_{1}$ represents the impact of baseline costs on total costs, ${\alpha }_{2}$ quantifies the association between costs and QALYs while ${\alpha }_{3}$ represents the effects of site.

QALYs are modelled as:

$$\begin{array}{ll}{e}_{i} \sim {\text{Normal}}\left({\mu }_{ei},{\sigma }_{e}^{2}\right)\\ {\mu }_{ei} ={\beta }_{0}+{\beta }_{1}\left({u}_{0i}-{\overline{u} }_{0}\right)+{\beta }_{2}{s}_{i}\end{array}$$

where the parameters ${\mu }_{ei}$ and ${\sigma }_{e}$ are the (individual-level) mean and the (population-level) standard deviation for QALY. In line with the original economic evaluation, centred version of utility values at baseline $\left( {u_{0i} - \overline{u}_{0} } \right)$ and site ${s}_{i}$ are included as covariates in the QALY model.

Suitable priors are specified for parameters to complete the model (see Appendix A for details and Appendix D for prior sensitivity assessment in the electronic supplementary material [ESM]); vague priors are assigned to the regression coefficients and Uniform priors are assumed for the standard deviations.

#### Gamma Model for Cost, Beta Model for QALYs

Since cost data are often right-skewed and non-negative, the cost component of the Normal model is expanded by assuming a Gamma distribution to reflect these features [23–25]. To facilitate prior specification and improve interpretability, the Gamma distribution for the costs given QALYs is re-parameterised by defining the shape parameter ${\zeta }_{ci}={\mu }_{ci}{\tau }_{ci}$ and the rate parameter ${\tau }_{ci}={\mu }_{ci}/{\sigma }_{c}^{2}$, where ${\mu }_{ci}$ and ${\sigma }_{c}$ describe the (individual-level) mean and the (population-level) standard deviation of the costs, respectively. This parameterisation allows priors to be placed directly on more intuitive parameters such as cost standard deviation and avoids the difficulty of formalising straightforward prior knowledge for the shape and rate parameters. A log link function is used to connect the conditional mean costs given QALYs and relevant covariates. Since some participants accrued zero costs in the trial, we re-scale the observed values for the costs by adding a small constant $\epsilon =1$ to the original data [8]. We note that modifying the data is necessary only to fit Gamma distributions to costs and has minimal impact on the conclusions under the overall scope of the study.

The costs conditional on the QALYs are modelled as:

$$\begin{array}{ll}{c}_{i}\mid {e}_{i} \sim {\text{Gamma}}\left({\mu }_{ci}{\tau }_{ci},{\tau }_{ci}\right)\\ \text{log}\left({\mu }_{ci}\right) ={\alpha }_{0}+{\alpha }_{1}\left({c}_{0i}-{\overline{c} }_{0}\right)+{\alpha }_{2}\left({e}_{i}-\overline{e }\right)+{\alpha }_{3}{s}_{i}\end{array}$$

The QALYs’ empirical distributions also show some degree of skewness and are theoretically restricted within the range $\left(0,0.5\right)$ in the ORBIT trial. Following the recommendation of previous research [26], a Beta distribution is fitted to the QALYs to capture the skewness of the outcome. The distribution of the QALYs is parameterised by two shape parameters, ${\kappa }_{ei}={\mu }_{ei}{\phi }_{ei}$ and ${\gamma }_{ei}=\left(1-{\mu }_{ei}\right){\phi }_{ei}$, where ${\mu }_{ei}$ represents the (individual-level) mean while ${\phi }_{ei}$ is an (individual-level) scale parameter, defined as:

$$\begin{array}{c}{\phi }_{ei}=\frac{\left(1-{\mu }_{ei}\right){\mu }_{ei}}{{\sigma }_{e}^{2}}-1\end{array}$$

Such parameterisation allows construction of the priors directly on the standard deviations of the QALYs and brings more intuitive understanding of the model. A logit link function is applied for the generalised linear model.

The model for the QALYs can be expressed as:

$$\begin{array}{ll}{e}_{i} \sim {\text{Beta}}\left({\mu }_{ei}{\phi }_{ei},\left(1-{\mu }_{ei}\right){\phi }_{ei}\right)\\ {\text{logit}}\left({\mu }_{ei}\right) ={\beta }_{0}+{\beta }_{1}\left({u}_{0i}-{\overline{u} }_{0}\right)+{\beta }_{2}{s}_{i}\end{array}$$

The model is completed by placing priors on the regression coefficients and the standard deviations: ${\text{Normal}}\left(0,{100}^{2}\right)$ for the coefficients in the cost model, ${\text{Normal}}\left(0,{2}^{2}\right)$ for the coefficients in the QALY model given the use of logit link; wide Uniform priors have been given to cost standard deviations—they have been selected to convey minimal information to the analysis and to ensure consistency with the Normal distribution choices for model comparisons throughout this study; although Uniform priors have been placed on the standard deviations of the QALYs, they are restricted using a ${\text{U}}{\text{niform}}\left(0,\sqrt{{\mu }_{e}\left(1-{\mu }_{e}\right)}\right)$ due to the property of Beta distribution [4].

#### Log-Normal Model for Cost, Beta Model for QALY

The model above is modified by assuming that the cost data are log-normally distributed, while maintaining the Beta distribution for the QALYs.

The cost Log-Normal model can be written as:

$$\begin{array}{ll}{c}_{i}\mid {e}_{i} \sim \text{Log-Normal}\left({\nu }_{ci},{\delta }_{c}^{2}\right)\\ {\nu }_{ci} ={\alpha }_{0}+{\alpha }_{1}\left({c}_{0i}-{\overline{c} }_{0}\right)+{\alpha }_{2}\left({e}_{i}-\overline{e }\right)+{\alpha }_{3}{s}_{i}\end{array}$$

It should be noted the ${\nu }_{ci}$ and ${\delta }_{c}$ in the Log-Normal model are now (individual-level) mean and (population-level) standard deviation of costs on the log scale, respectively. To obtain the mean (${\mu }_{ci}$) and standard deviation (${\sigma }_{ci}$) of costs on their original scale for each individual $i$, the following expressions could be used:

$$\begin{array}{ll}{\mu }_{ci} =\text{exp}\left({\nu }_{ci}+\frac{{\delta }_{c}^{2}}{2}\right)\\ {\sigma }_{ci} =\sqrt{\left[\text{exp}({\delta }_{c}^{2})-1\right]\text{exp}\left(2{\nu }_{ci}+{\delta }_{c}^{2}\right)}\end{array}$$

Similar to the Gamma distribution, the Log-Normal distribution requires the data to be positive, necessitating the addition of a small value ($\epsilon =1$) to the original cost data. Vague Normal prior distributions are assigned to regression coefficient parameters.

Uniform priors with different upper bounds are considered for the standard deviations of log costs in Log-Normal distribution (see Appendix A in the ESM) to assess their impact on the cost-effectiveness results. Specifically, to ensure the comparability across models with different distributional assumptions, the Uniform prior distributions on the log-cost standard deviation in the Log-Normal models are intended to lead to reasonable and comparable ranges to those on the original cost scale in other models. The exact upper bound values to be considered in the Log-Normal model may depend on the mean of the data, given the mathematical properties of the Log-Normal distribution. When the actual mean costs are large, a Uniform prior distribution with a large upper bound on log-cost standard deviation may produce unrealistic results. An illustration of the potential impact of the Uniform priors for log-scale standard deviation on original-scale standard deviation is provided in Appendix B (see ESM). Therefore, Uniform prior distributions with narrower and reasonable ranges have been chosen for the Log-Normal models, compared with those in the Normal and Gamma models.

### Implementation

All models are fitted in JAGS [27], a program for Bayesian inference based on Markov Chain Monte Carlo (MCMC) via the R2jags package in R version 4.0.3 [28]. We run two chains, each with 52,000 iterations, and discard the first 2000 iterations from each chain as a burn-in phase. A thinning rate of 10 has also been applied to reduce autocorrelation, leading to a total sample of 10,000 iterations for inference. Convergence is assessed through potential scale reduction statistics, $\widehat{R}$ [29], and a visual inspection on trace plots. To overcome the difficulty in model convergence arising from the differing scales of costs and QALYs, the original cost data have been scaled down by a factor of 10, but the resulting inferences are robust to the choice of the scaling factor. Model fit is assessed through posterior predictive checks (see Appendix C in the ESM) and compared by deviance information criterion (DIC) [30].

Given the main focus of our study, cost estimates are initially reported for the CEA models to identify how much the marginal mean costs can be affected by different upper bounds of the Uniform distributions. Mean costs with different distributional assumptions are also compared to investigate whether the influence brought by the Uniform prior distributions on cost standard deviations is sensitive to the choice of cost distributions. After that, the impact of using Uniform prior distributions on cost-effectiveness results is explored using a cost-effectiveness plane [31] and cost-effectiveness acceptability curve (CEAC) [32].

## Results

### ORBIT Trial Data

**Fig. 1:**
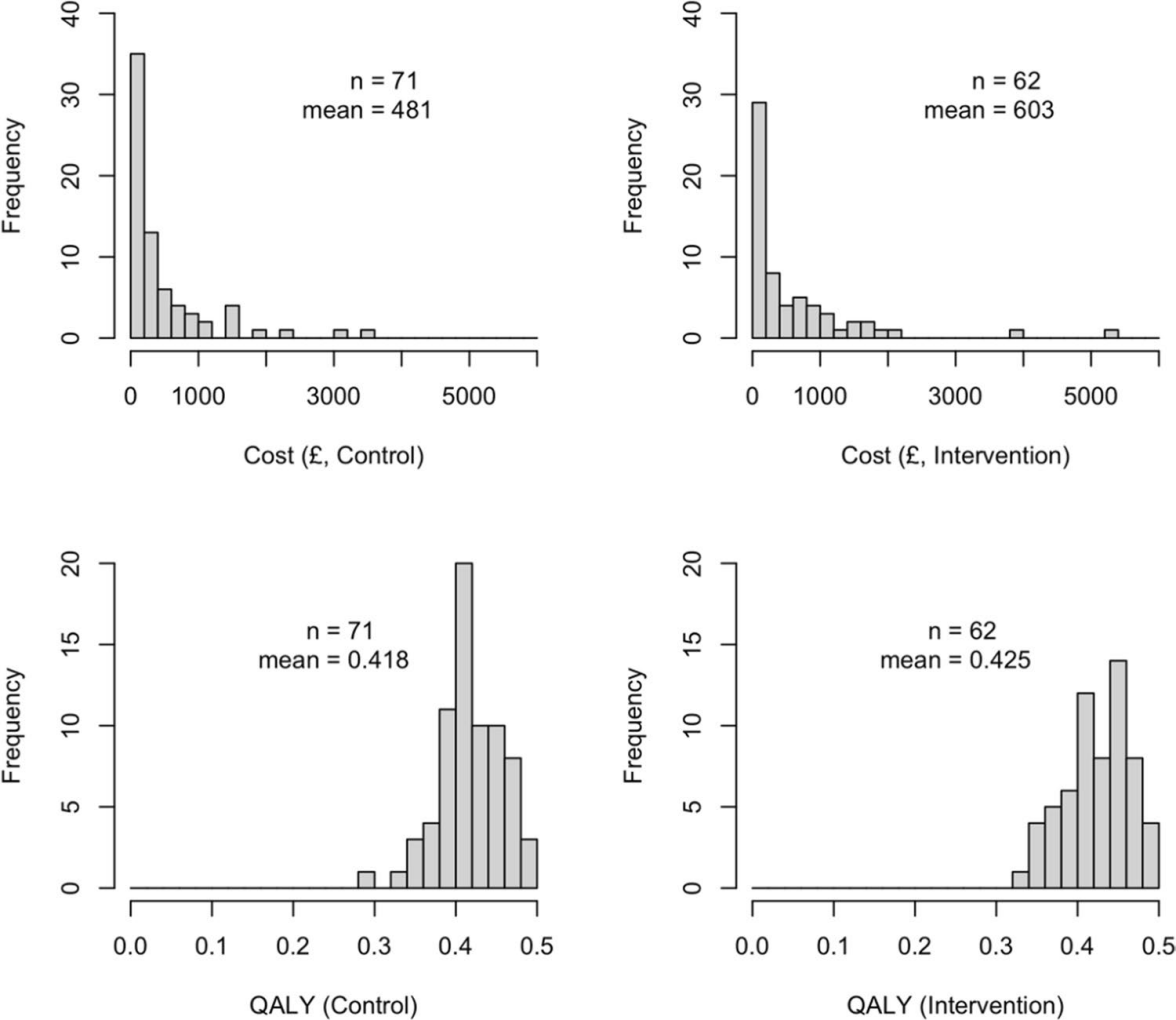
Distribution of trial data by treatment arms. *QALY* quality-adjusted life years

Both costs and QALYs show some degree of skewness (Fig. 1). Most of the cost data are below £2000 with three participants as an exception in each treatment arm. A comparable proportion of participants with zero health care costs in both treatment arms is observed: 5/62 (8.06%) participants accrue zero costs in the intervention while 6/71 (8.45%) participants do so in the control. QALYs mostly lie between 0.3 and 0.5 with no participant having a perfect health status of 0.5 by the end of the 6-month follow-up.

### Cost Analysis

The specification of a Uniform distribution as the prior on standard deviation for log costs in the Log-Normal model influences results when zero values exist in the cost data (Table 1). Compared with the Normal and Gamma models, the model fit and cost estimates from the Log-Normal models are more sensitive to the choice of upper bounds for the Uniform priors. The Log-Normal models with the two lowest upper bound values for the Uniform prior present higher DIC values than the others, suggesting a poorer fit to the data, which has also been confirmed via posterior predictive check (see Appendix C in the ESM). Improvements in model fit can be observed when the upper bound of the Uniform prior distribution increases. Although the DICs of the Log-Normal models with ${\text{Uniform}}\left(0,2\right)$ and ${\text{Uniform}}\left(0,3\right)$ are similar, the two models produce different scales of cost estimates: the incremental mean costs of the intervention compared with the control are £344 (95% credible intervals: −2258, 3256) with ${\text{Uniform}}\left(0,2\right)$ but they jump to £1301 (95% CIs: −4169, 8699) when ${\text{Uniform}}\left(0,3\right)$ is applied. The Normal models do not fit the data well, as their DIC values are higher than those of the Gamma models and most Log-Normal models. In contrast, the Gamma models show the best fit to the data, as confirmed by both DIC and posterior predictive checks.

**Table 1:** Marginal mean and incremental mean cost estimates, with 95% credible intervals in parentheses, for models with different uniform prior distributions on cost standard deviations

Prior	DIC	Costs (Con)		Costs (Int)		Incremental costs	
*Normal model*							
Uniform (0, 1000)	948.8	476	(320, 632)	598	(421, 775)	123	(−102, 366)
Uniform (0, 10000)	948.8	476	(320, 632)	598	(421, 775)	123	(−102, 366)
*Gamma model*							
Uniform (0, 1000)	718.7	537	(388, 692)	709	(451, 970)	171	(−128, 484)
Uniform (0, 10000)	718.4	535	(393, 697)	710	(469, 982)	174	(−131, 480)
*Log-normal model*							
Uniform (0, 3)	744.4	2095	(497, 4761)	3396	(620, 8279)	1,301	(−4169, 8699)
Uniform (0, 2)	740.1	1610	(489, 3266)	1954	(651, 3868)	344	(−2258, 3256)
Uniform (0, 1)	919.3	412	(260, 585)	468	(287, 667)	56	(−208, 335)
Uniform (0, 0.8)	1126.9	337	(241, 453)	386	(265, 518)	49	(−127, 215)

Costs are measured using British pounds (£)

*Con* control, *DIC* deviance information criteria, *Int* intervention

**Fig. 2:**
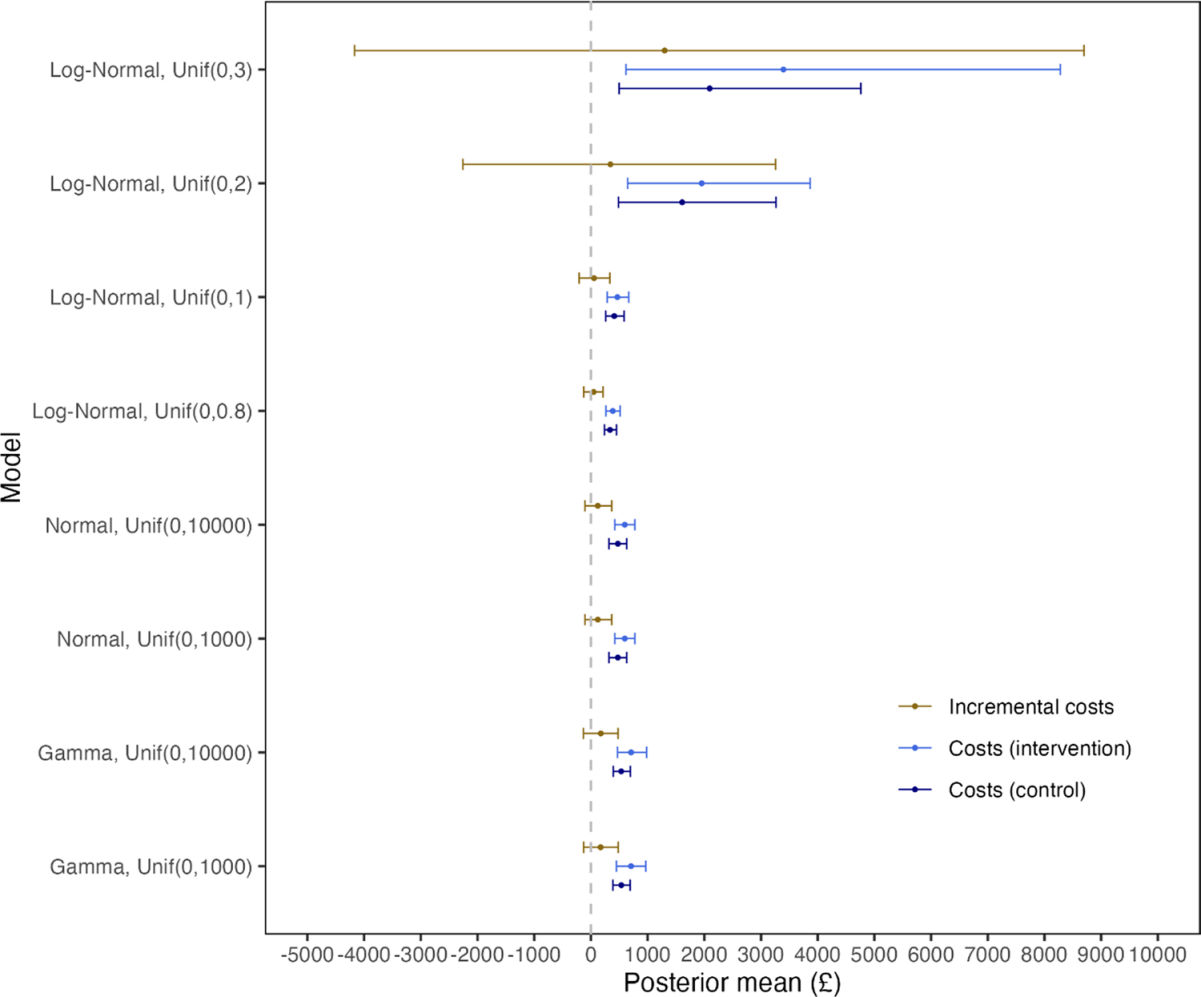
Marginal mean cost by treatment arms, incremental mean costs and their 95% credible intervals of Normal, Gamma and Log-Normal models with different Uniform prior distributions on cost standard deviations

Mean cost estimates by treatment arms, incremental mean costs and their 95% CIs are graphically compared across models with different distributional assumptions and prior distributions (Fig. 2). Unlike with the Normal and Gamma models, the Log-Normal model exhibits large variations in cost estimates based on the choice of the Uniform priors for standard deviations of log costs. The use of ${\text{Uniform}}\left(0,0.8\right)$ and ${\text{Uniform}}\left(0,1\right)$—the two Uniform priors associated with a poorer fit to the data—results in comparable cost estimates to those produced from the Normal and Gamma models. In contrast, the other two Uniform priors, which have shown a better model fit, can inflate the mean cost estimates in the two treatment arms (and thus incremental mean costs), even when the upper bound of the Uniform prior distribution has been limited to 2. The Normal and Gamma models are more robust to the choice of upper bounds of the Uniform priors compared with the Log-Normal model, producing similar results across the different upper bound values explored.

### Cost-Effectiveness Results

**Fig. 3:**
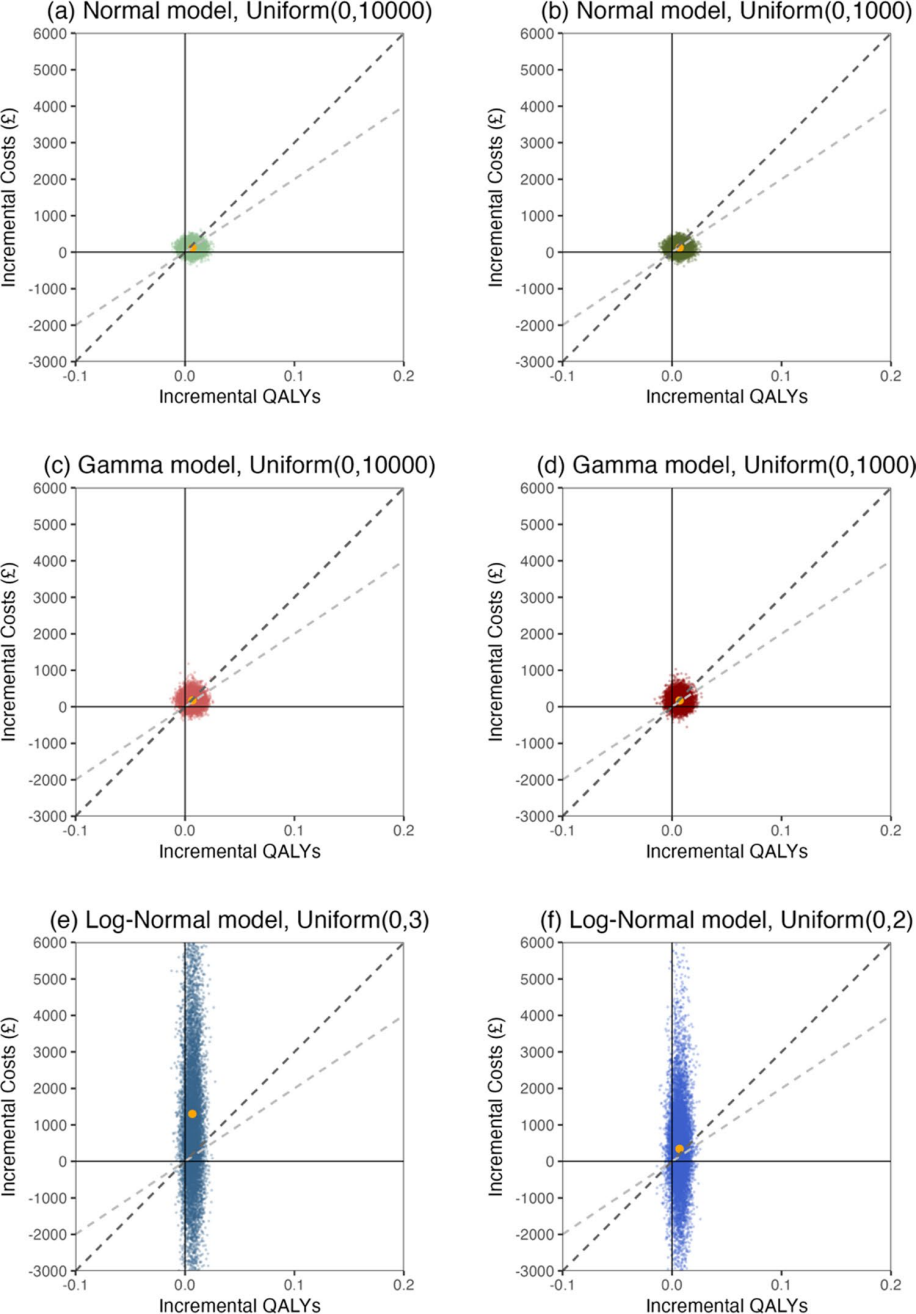
Cost-effectiveness planes across Normal, Gamma and Log-Normal models with Uniform priors for cost standard deviations. The *grey dashed line* represents a willingness-to-pay threshold at £20,000 while the *black dashed line* represents a threshold at £30,000. The *orange dot* in each sub-figure denotes the incremental cost-effectiveness ratio (ICER) of each model. The Uniform priors are placed on standard deviation of original costs in Normal and Gamma models while allocated to standard deviations of log costs in Log-Normal models. *QALYs* quality-adjusted life years

Figure 3 shows the cost-effectiveness planes of models based on different distributional assumptions and varying Uniform priors for cost standard deviations. Here we specifically focus on the cost-effectiveness planes for the two Uniform distributions with a better fit to the data—${\text{Uniform}}\left(0,2\right)$ and ${\text{Un}}{\text{iform}}\left(0,3\right)$—as priors on the standard deviations of log costs in Log-Normal models. The decision is made because the ${\text{Uniform}}\left(0,0.8\right)$ and ${\text{Uniform}}\left(0,1\right)$ (though their cost-effectiveness planes show similar patterns to Normal and Gamma models) appear too restrictive for the data based on both their DIC values and posterior predictive checks.

The cost-effectiveness planes illustrate how sensitive the cost-effectiveness results are to the choice of models and upper bounds of the Uniform prior distributions for cost standard deviations. The pairs of incremental costs and QALYs, and the associated incremental cost-effectiveness ratio (ICER), predominantly fall below the willingness-to-pay threshold of £30,000 in the Normal and Gamma models, supporting the cost-effectiveness of the intervention. Conversely, the Log-Normal models present much higher incremental costs and ICERs compared with models with other distributional assumptions.

The sensitivity of the cost-effectiveness results to the upper bounds of the Uniform priors may be affected by model distributional assumptions. As shown in Fig. 3, panels (a) and (b) for the Normal model, and panels (c) and (d) for the Gamma model, the two models are robust to the upper bounds of the Uniform prior distributions for their cost standard deviations. In contrast, the results of the Log-Normal models can be greatly influenced by the Uniform prior distributions used for the standard deviations of log costs, as illustrated in Fig. 3, panels (e) and (f). An increase in the upper bounds of the Uniform priors can bring more extreme values and substantial uncertainty to the incremental mean costs, thus becoming less likely to conclude that intervention is cost-effective.

**Fig. 4:**
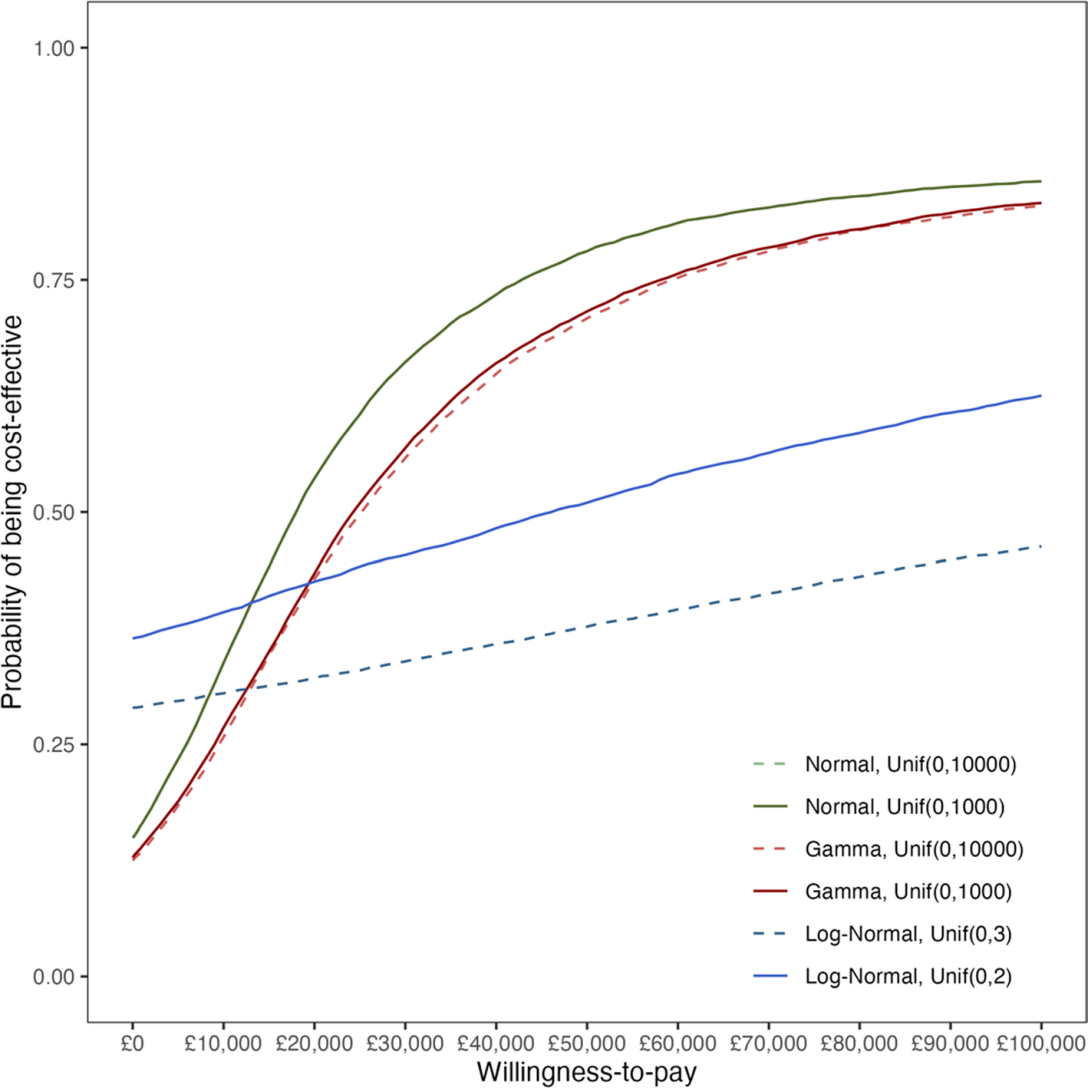
Cost-effectiveness acceptability curves across Normal, Gamma and Log-Normal models with different Uniform prior distributions. The *solid lines* indicate results using Uniform priors with narrower ranges, i.e. ${\text{Uniform}}\left(\text{0,1000}\right)$ for the Normal and Gamma models and ${\text{Uniform}}\left(\text{0,2}\right)$ for the Log-Normal model; while the *dashed lines* represent results based on Uniform priors with wider ranges, i.e., ${\text{Uniform}}\left(\text{0,10000}\right)$ for the Normal and Gamma models and ${\text{Uniform}}\left(\text{0,3}\right)$ for the Log-Normal model. The *green lines* show CEACs from the Normal models, the *red lines* are for the Gamma models and the *blue lines* are for the Log-Normal models. The CEACs for the two Normal models overlap due to the wide Uniform priors placed on the cost standard deviations on the original scale

The CEACs show that the intervention is more likely to be cost-effective under the Normal and Gamma models compared with the Log-Normal models when a willingness-to-pay threshold reaches £30,000 or above (Fig. 4). When Uniform prior distributions are used for the log cost standard deviations in the Log-Normal models, the uncertainty in incremental mean costs reduces the probability of the intervention being cost-effective. As the upper bound of the Uniform prior distribution decreases to 2, the probability that the intervention is more cost-effective than the control increases.

## Simulations

### Simulation Overview

Building upon the findings of the case study, a simulation study was designed to further evaluate the performance of the Bayesian CEA models (i.e. Normal, Gamma and Log-Normal models with different Uniform prior distributions on cost standard deviations). We investigated the impact of these models and prior choices on cost estimates in CEAs, including the mean cost estimates for each treatment arm and their incremental costs. The aim of this simulation study was to explore how sensitive the Bayesian CEA cost models are to the choice of priors for cost standard deviations and how this sensitivity might vary across different scenarios.

The simulation settings were designed to reflect the common challenges that might be encountered in real-world CEA applications, motivated by our case study. We considered a cost-effectiveness analysis alongside a simple, 6-month, two-arm RCT when generating individual-level cost-effectiveness data. In this simulation study, we assumed that baseline utilities and QALYs are normally distributed, however, this choice did not influence the conclusions of the entire study as we focused on the impact of prior distributions on cost estimates.

### Data Generating Process

Cost data are often skewed and prone to zero values, indicating no use of health care services. The findings from the case study suggest that the occurrence of zero values in cost data may lead to poor performance of the Log-Normal cost model; however, the impact of the proportion of zeros on its relative performance remains unclear. Additionally, Bayesian analyses are known to be sensitive to the prior specification when sample sizes are small [33]. Therefore, a total of eight scenarios were defined based on the three dimensions on cost—data skewness, proportion of zeros and sample size.

- Data skewness: To account for different levels of skewness, the positive values in cost data are assumed to follow Gamma and Log-Normal distributions, respectively.
- Proportion of zero values: Scenarios with relatively low proportions of zeros (varying between 0% and 10%) are considered to mimic real-world situations with either no or mild occurrences of zeros in cost data. For cases with higher proportions of zero values, a two-part model is recommended [34].
- Sample size: The total sample size is set to either 200 or 2000, representing 100 and 1000 participants per treatment arm, respectively, under the assumption of a 1:1 allocation rate between intervention and control groups. The sample size of 200 reflects the situation typified by the ORBIT trial, while the larger sample size of 2000 represents a typical high-sample-size scenario for trial-based CEAs.

Initially, QALYs were generated from a Normal distribution and then cost data were simulated from a two-part model to determine the presence of zero values. Further details of the data generating process are provided in Appendix E due to space limitations (see ESM).

We conducted 1000 simulations for each scenario and fit three CEA models with different Uniform priors using a Bayesian approach to align with the case study and allow a fair comparison within the simulation setting. Again, a constant value ($\epsilon =1$) was added to the Gamma and Log-Normal models in scenarios with zeros. The QALY model component in the Bivariate Normal model was retained consistently for the Gamma and Log-Normal models.

The focus of this simulation was the estimation of mean cost per treatment arm and incremental costs. To assess the relative performance in terms of treatment-specific mean costs and incremental costs across the Normal, Gamma and Log-Normal models with different Uniform priors on cost standard deviations, we compared their bias, empirical standard error (Empirical SE) and root mean squared error (RMSE) in each simulated scenario. The definitions of these performance measures are provided in Appendix E (see ESM).

The Log-Normal distribution with Uniform priors whose ranges are more limited, that is, Uniform (0, 1) and a Uniform (0, 0.8), cannot converge when cost data with a large sample size of 2000 follow a Gamma distribution and present no zeros. Data re-scaling has not been considered for this scenario as it may distort the original relationship between the data scale and the choice of prior, which does not align with the initial purpose of this study. Therefore, the Log-Normal models with these two Uniform priors will not be reported in this scenario.

### Simulation Results

**Fig. 5:**
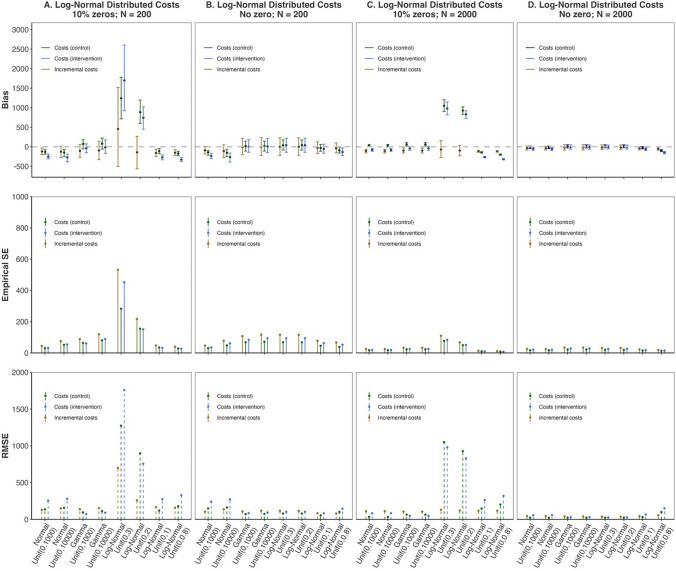
Relative performance of the Normal, Gamma and Log-Normal models with different Uniform prior distributions when cost data are log-normally distributed. Each sub-figure reports the performance of the models in terms of their bias (with its 95% credible intervals), empirical standard error (SE) and root mean squared error (RMSE) arranged from top to bottom, for the estimation of mean costs per treatment arm and incremental costs. Column A of sub-figures presents model performance under scenarios with 10% zero values and a small sample size of 200, while column B shows scenarios with no zero values and the small sample size. The remaining two columns (C and D) report model performance for scenarios with a larger sample size of 2000

Figure 5 graphically summarises the performance of the assessed models across different distributional assumptions and Uniform prior distributions for cost standard deviations when cost data are log-normally distributed. As expected, when cost data follow a Log-Normal distribution, the occurrence of zeros strongly undermines the performance of the Log-Normal models, regardless of the sample size (as shown in Fig. 5, Column A and C). Under these scenarios, the Log-Normal model is highly sensitive to the upper bound values of the Uniform priors for cost standard deviation, influencing the mean cost estimates in terms of more bias in each treatment arm.

In scenarios without zeros, the Log-Normal model still exhibits sensitivity to the prior selection. Specifically, estimates from Log-Normal models under wider Uniform priors—${\text{Uniform}}\left(0,2\right)$ and ${\text{Uniform}}\left(0,3\right)$—are less biased than those using narrower priors—that is, ${\text{Uniform}}\left(0,1\right)$ and ${\text{Uniform}}\left(0,0.8\right)$. This finding suggests that the narrower Uniform prior may be overly restrictive for these scenarios. However, the Log-Normal models show robustness when the two wider Uniform prior distributions are used.

The Normal and Gamma models seem to present a higher degree of robustness to their prior specifications compared with the Log-Normal models. When there is no zero in the data, the Normal models have greater bias and RMSE than the Log-Normal models with wider Uniform priors, irrespective of sample size. The Normal model performance improves compared with the Log-Normal models with narrower Uniform priors when the sample size is large. Surprisingly, the Gamma models achieve comparable performance to that of the Log-Normal models with wider Uniform prior distributions, even when data are log-normally distributed and are fully positive.

In this simulation study, when the cost data follow a Gamma distribution, the relative performance of the Log-Normal models is inferior to the other models. Taking the mean bias in incremental costs as an example, as illustrated in Table 2, the Log-Normal models show greater bias and variations to their prior selections compared with the Normal and Gamma models, especially when the data contain no zero values. This is likely due to the Gamma distribution being able to generate small cost values close to zero, without the need to add a small constant to the original data when fitting the Log-Normal models in these scenarios. Given this mismatch between the true underlying cost distribution and the Log-Normal model specification, and considering the presence of near-zero values, the performance of the Log-Normal models deteriorates substantially and becomes extremely sensitive to the choice of priors.

**Table 2:** Bias in incremental costs of Normal, Gamma and Log-Normal models with different uniform prior distributions when cost data follow a Gamma distribution

Models	Scenarios			
	10% zeros *N* = 200	No zero *N* = 200	10% zeros *N* = 2000	No zero *N* = 2000
*Normal model*				
Uniform (0,1000)	−127	−117	−116	−81
Uniform (0,10000)	−140	−134	−117	−82
*Gamma model*				
Uniform (0,1000)	−91	−80	−120	−66
Uniform (0,10000)	−56	−33	−110	−62
*Log-Normal model*				
Uniform (0,3)	459	977,821	−146	27,069
Uniform (0,2)	−320	68,408	−676	1307
Uniform (0,1)	−171	13,904	−250	NA
Uniform (0,0.8)	−162	11,497	−229	NA

In contrast, both Normal and Gamma models are more robust to the Uniform prior specification. They are generally less biased and more precise than the Log-Normal models across all four Gamma-distributed data scenarios. Full details on model relative performance, including bias in mean costs per treatment arm, empirical SE and RMSE, are provided in Appendix E (see ESM), given that they are similar to the findings for bias in incremental costs reported in Table 2.

## Discussion

This study compares the impact of implementing Uniform prior distributions with different upper bounds on CEA results under a Bayesian modelling framework. These Uniform prior distributions are applied on log-scale cost standard deviations when costs are modelled using a Log-Normal distribution, and on original cost standard deviations when costs are assumed to follow a Normal or Gamma distribution. The analysis is initially based on a real case study with a non-negligible proportion of patients having zero costs and then validated via a simulation approach. Our study shows that Bayesian CEAs can be sensitive to the choice of upper bounds of the Uniform prior distributions for log cost standard deviations when cost data contain zero values and are assumed to be log-normally distributed. Unlike the Log-Normal distribution, the Normal and Gamma distributions have shown robustness to the upper bounds of Uniform distributions as the priors on the standard deviations of costs.

It is common to use Uniform distributions as the priors on standard deviations of cost data in Bayesian CEAs due to their benefits of conveying minimal information through the priors when limited knowledge about these parameters is available. The underlying belief is that given the lack of knowledge on variance parameters, the bounds for ‘minimally informative’ Uniform priors are not expected to influence final conclusions substantially [35]. However, our study indicates the importance of recognising the appropriateness of Uniform prior distributions in applied Bayesian CEAs. The use of such priors can potentially bring ‘unwanted’ information into the posterior inference and lead cost estimates towards unintended values.

One potential reason for the overly large cost estimates found in our study is that the Uniform prior distribution is applied on the standard deviation of costs on the log scale rather than on the original scale in the Log-Normal distribution. Indeed, concerning the mathematical property of the Log-Normal distribution, when a Uniform prior distribution is assigned to the standard deviation of log costs, the magnitude of costs on its original scale can, possibly, explosively increase to an extreme high level. In other words, even small upper bounds of Uniform priors can yield large standard deviations on original costs and thus lead to mean cost estimates that are unlikely to occur in reality. Using Uniform distribution as a minimally informative prior for the purpose of letting data dominate posterior inference is clearly not effective in most of the scenarios explored in our analysis.

Another possible explanation is the characteristics of the actual data. Our case study features some typical characteristics of CEA data that may contribute to the poor performance of the Log-Normal model, which potentially drives the sensitivity of cost-effectiveness results to the Uniform prior distributions. These include small sample sizes, potential model misspecification for skewed data and the existence of zero-cost observations. Our simulation shows that, if the data contain zero, the cost-effectiveness results would remain substantially sensitive to Uniform priors, even when sample size is increased and regardless of whether the data are generated from a Log-Normal or Gamma distribution. This suggests that the sample size and model misspecification are less likely to be the primary driver of the sensitivity observed. Surprisingly, the performance of the Log-Normal model is extremely unreliable when cost data follow a Gamma distribution. This aligns with a previous simulation study that investigates parametric modelling options for costs based on data generated from parametric distributions [25].

The excessively large cost estimates from our empirical analysis of trial data may be related to the existence of several observations with zero costs over time. In line with our case study, a previous study that focuses on Bayesian cost prediction using cohort data with excess zeros has also found that Log-Normal models could overestimate mean observed costs [36]. To date, very few Bayesian CEAs using Log-Normal distribution for costs report whether they encounter the same situation where a non-negligible proportion of zero occurs in the cost data. Our simulation study provides evidence that cost estimates—biased with the presence of zero values—can be unbiased and insensitive to appropriate Uniform prior distributions when all cost values are strictly positive. A related study that explores various modelling approaches to handle zero-cost data via a simulation approach supports our finding, highlighting the poor performance of the Log-Normal model under low, moderate and high proportions of zeros [34].

Based on the aforementioned results, we recommend that the sensitivity of cost-effectiveness results to Uniform prior distributions with different upper bounds is assessed in any CEA involving individual-level zero cost data. A conservative alternative is to rely on models that are more robust to the upper bounds of Uniform prior distributions, for example, the Gamma model, or those that can address the zero-cost feature, such as a hurdle model (also referred as a two-part model) [12, 35].

Despite the wide use of Bayesian methods in CEAs, no guidance exists that provides validated advice on prior elicitation from a statistical perspective, nor does it clearly illustrate the possible consequences resulting from the choice of prior distributions. Our study attempts to explore the influence of Uniform distributions as minimally informative prior distributions on standard deviations of costs in a CEA context through both a case study and a simulation approach. Limitations of our analysis include the following: first, the simulation study has not addressed whether and how much the poor performance of the Log-Normal model, and its sensitivity to the Uniform prior distributions, are influenced to the continuity correction for zero values, that is, adding a constant of one to the original data. As a result, we could not explore any potential remedies for the issues introduced by CEA data containing zeros. Second, we did not compare different choices of minimally informative prior distributions and thus cannot make evidence-based recommendations for future applications. However, the current study structure—starting with an empirical analysis of real-world trial data and followed by a tailored simulation study—would be sufficient to support our primary goal, that is, to reveal the potential impact of choosing Uniform prior distributions with large upper bounds as the log cost standard deviations of Log-Normal models in real applications.

## Conclusion

Bayesian CEAs are sensitive to the choice of upper bounds of the Uniform prior distributions for standard deviations of log costs when they are assumed to follow a Log-Normal distribution. Our results suggest careful consideration should be given to the potential consequences of applying Uniform distributions on cost standard deviations as minimally informative priors based on both distributional model assumptions and actual characteristics of the data. It may be more conservative to consider alternative minimally or weakly informative priors to assess the robustness of posterior results.

## Supplementary Information

Below is the link to the electronic supplementary material.

Supplementary file1 (DOCX 45437 KB)

## Declarations

### Data availability

The ORBIT trial data that support the findings of this article are not openly available from the authors due to a data access agreement with the original trial team. For access to the data, please contact the ORBIT trial team directly.
