# Supplementary material for: Bayesian Cost-Effectiveness Analysis Using Individual-Level Data is Sensitive to the Choice of Uniform Priors on the Standard Deviations for Costs in Log-Normal Models
Source: Pharmacoeconomics. 2025 Aug 12;43(11):1309–21. doi: 10.1007/s40273-025-01511-1 (PMC12534244; doi:10.1007/s40273-025-01511-1)
Supplement: Supplementary file 1 — Supplementary file1 (DOCX 45437 KB) [file 40273_2025_1511_MOESM1_ESM.docx]

## **Appendix**

This is the appendix for the article “Bayesian cost-effectiveness analysis is sensitive to the choice of Uniform priors on the standard deviations for costs in Log-Normal models”, submitted for publication in the journal *PharmacoEconomics*. This document is created by Xiaoxiao Ling from Nuffield Department of Primary Care Health Science at University of Oxford, UK ([xiaoxiao.ling@phc.ox.ac.uk](mailto:xiaoxiao.ling@phc.ox.ac.uk)).

## Appendix A. Prior Distributions for Bayesian Cost-Effectiveness Models

**Table 3** Prior Distributions for Bayesian Cost-Effectiveness Models

| Parameter | Normal model | Log Normal model | Gamma model |
| --- | --- | --- | --- |
| Coefficients in cost model ($\alpha_{j}$) | Normal(0, ${100}^{2}$) | Normal(0, ${100}^{2}$) | Normal(0, ${100}^{2}$) |
| Standard deviation, costs ($\sigma_{c}$) | Uniform(0, 1000) |  | Uniform(0, 1000) |
|  | Uniform(0, 10000) |  | Uniform(0, 10000) |
| Standard deviation, log costs ($\delta_{c}$) |  | Uniform(0, 3) |  |
|  |  | Uniform(0, 2) |  |
|  |  | Uniform(0, 1) |  |
|  |  | Uniform(0, 0.8) |  |
| Coefficients in QALY model ($\beta_{k}$) | Normal(0, ${100}^{2}$) | Normal(0, $2^{2}$) | Normal(0, $2^{2}$) |
| Standard deviation, QALYs ($\sigma_{e}$) | Uniform(0, 1000) | Uniform(0, $\sqrt{\mu_{e}\left( 1-\mu_{e} \right)}$) | Uniform(0, $\sqrt{\mu_{e}\left( 1-\mu_{e} \right)}$) |

*j = 0,1,2,3; k = 0,1,2;* $\mu_{e}$ *denotes mean QALYs.*

## Appendix B. Kernal density estimation of Uniform prior distributions on log-scale standard deviations in Log-Normal model against the original-scale standard deviations

Fig. 6 presents the kernel density estimation of the Uniform priors against the original-scale standard deviation. The figure illustrates the implications of Uniform prior distributions with different upper bounds on log-scale standard deviations for original-scale standard deviations in the Log-Normal model. The calculation of original-scale standard deviation in the Log-Normal distribution requires assumptions about the log-scale mean. However, an original-scale mean is more intuitive than a log-scale mean for the purpose of prior specification in a health economics context. Therefore, assumptions are made based on the original-scale mean. The subfigures in Fig. 6 are plotted from left to the right, assuming actual mean costs of £500, £1,000 and £2,000, respectively.


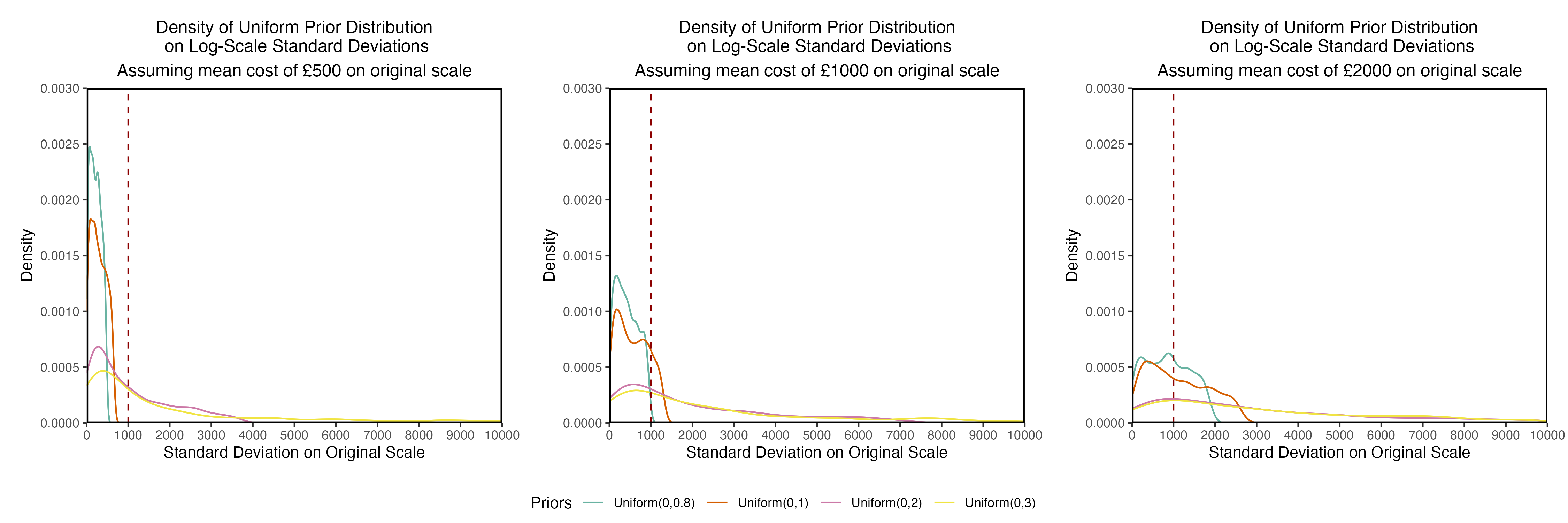


**Fig. 6** Kernel density estimation of Uniform prior distributions on log-scale standard deviations in Log-Normal model against the original-scale standard deviations. As the mean cost on the original scale increases, the same Uniform prior distributions on log-scale standard deviations imply a higher probability of larger standard deviations on the original scale.

## Appendix C. Posterior Predictive Checks

Fig. 7 to Fig. 14 show direct graphical posterior predictive checks for models with different distributional assumptions and prior specification for cost standard deviations. Replicated total health care costs and QALYs by treatment arm are generated from the posterior predictive distribution of the models, and compared to the distribution of the observed data. No systematic difference between replicated and observed data are expected if the model presents a good fit. However, it is obvious that the Normal models can not fit the cost data well.


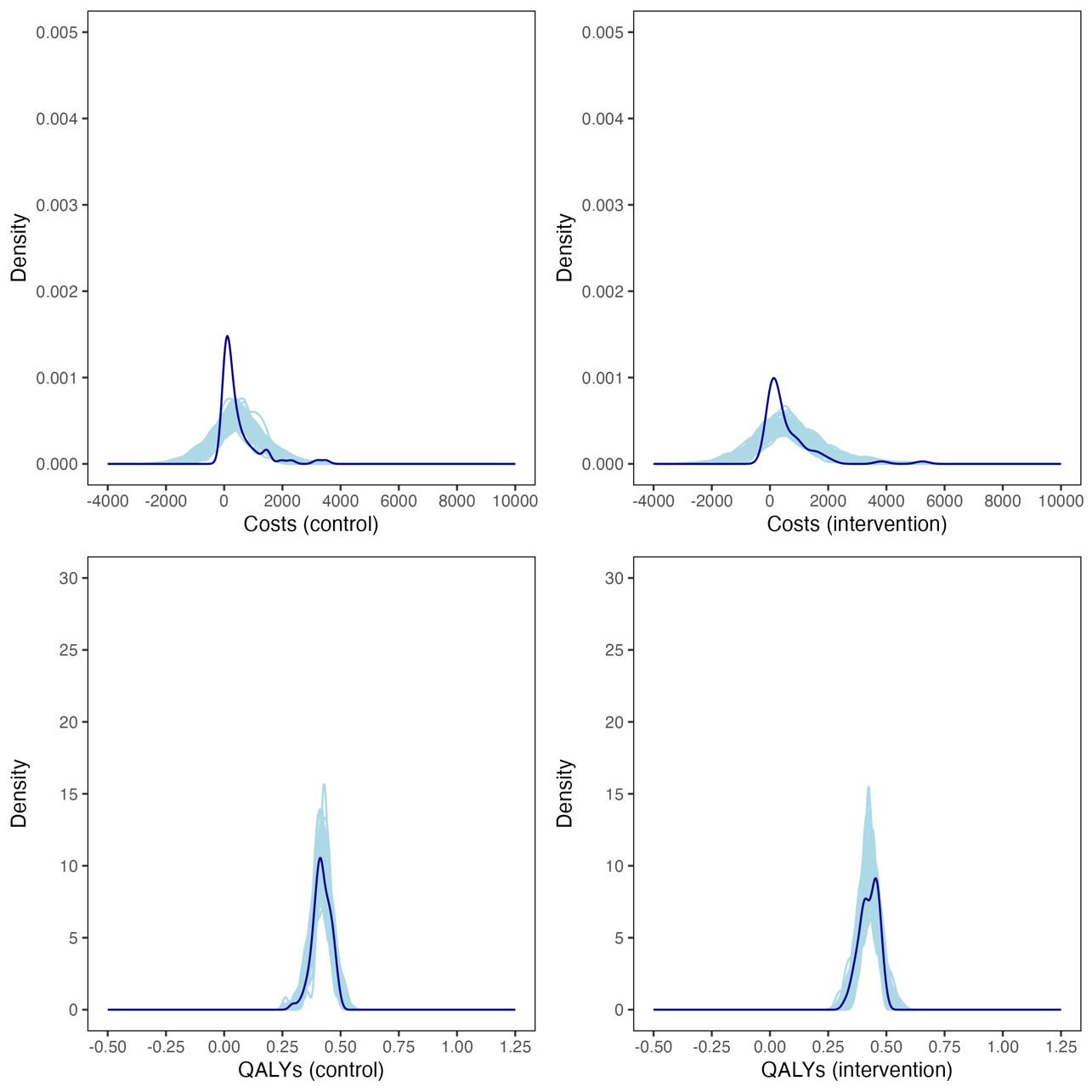


**Fig. 7** Distributions of replicated total health care costs and QALYs by treatment arm drawn from posterior predictive distribution compared to the distribution of observed data under the Normal model with Uniform(0,1000) as the prior on cost standard deviations. The dark blue curve represents observed data while the light blue curves display 100 simulated total health care costs and QALYs drawn from their posterior predictive distributions.


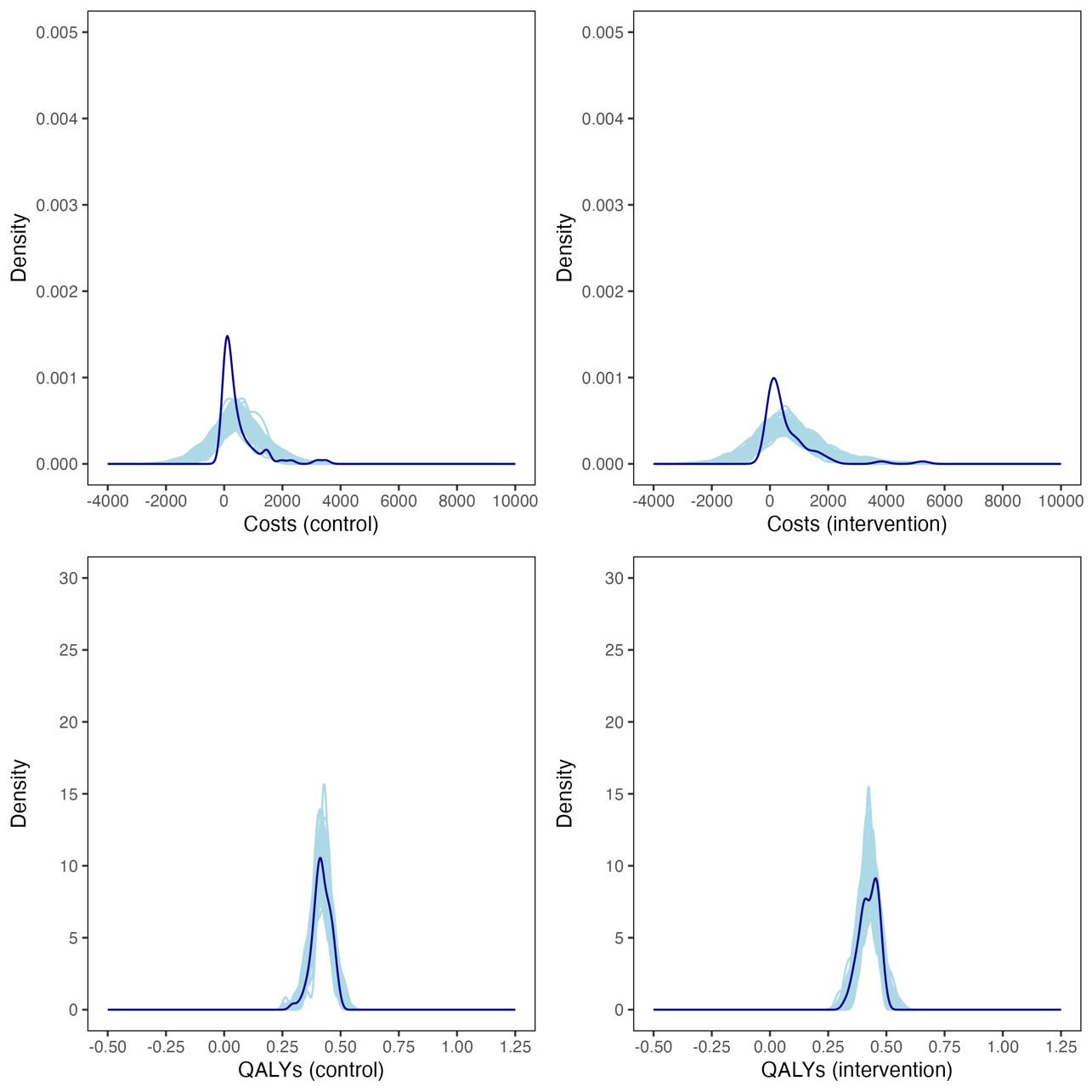


**Fig. 8** Distributions of replicated total health care costs and QALYs by treatment arm drawn from posterior predictive distribution compared to the distribution of observed data under the Normal model with Uniform(0,10000) as the prior on cost standard deviations. The dark blue curve represents observed data while the light blue curves display 100 simulated total health care costs and QALYs drawn from their posterior predictive distributions.


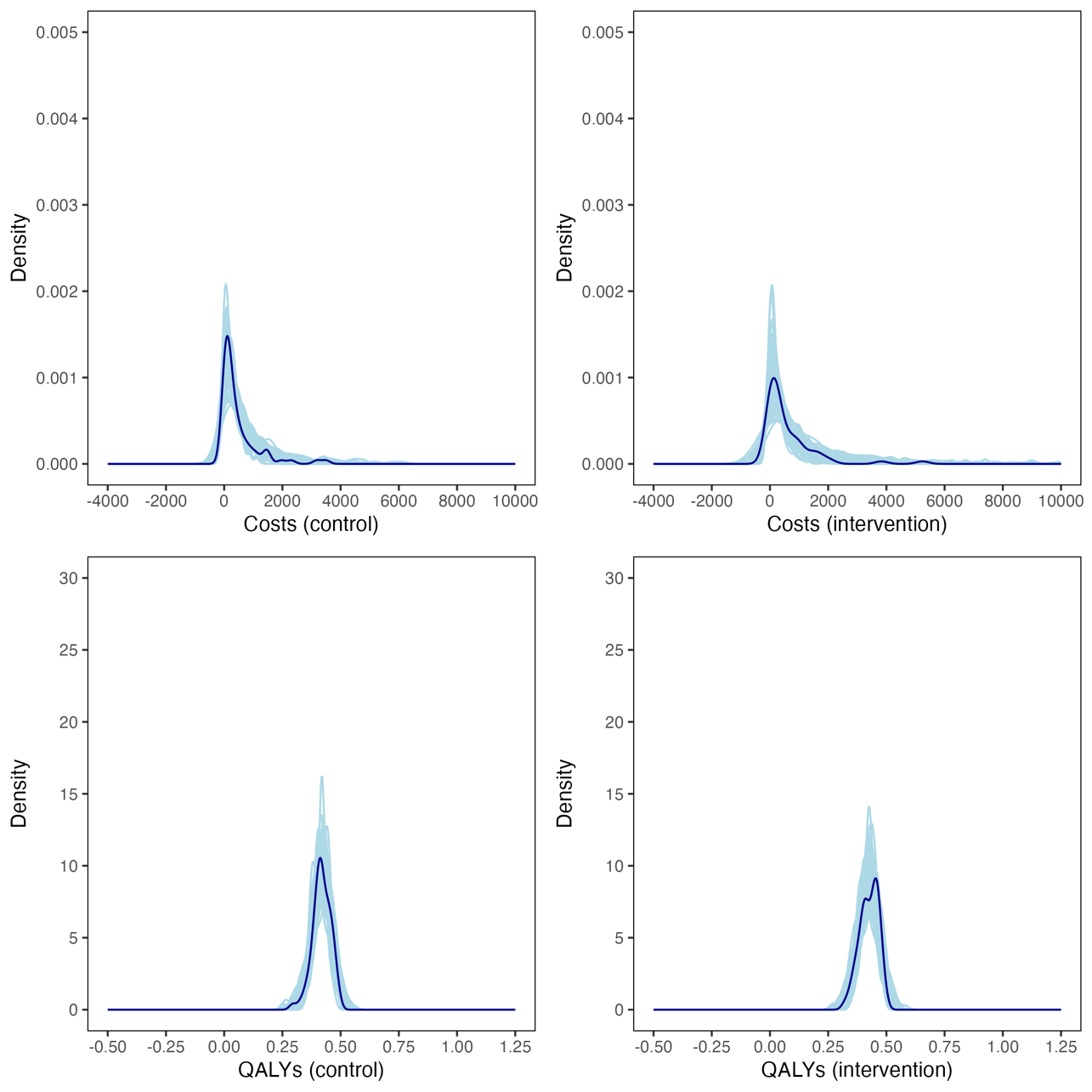


**Fig. 9** Distributions of replicated total health care costs and QALYs by treatment arm drawn from posterior predictive distribution compared to the distribution of observed data under the Beta Gamma model with Uniform(0,1000) as the prior on cost standard deviations. The dark blue curve represents observed data while the light blue curves display 100 simulated total health care costs and QALYs drawn from their posterior predictive distributions.


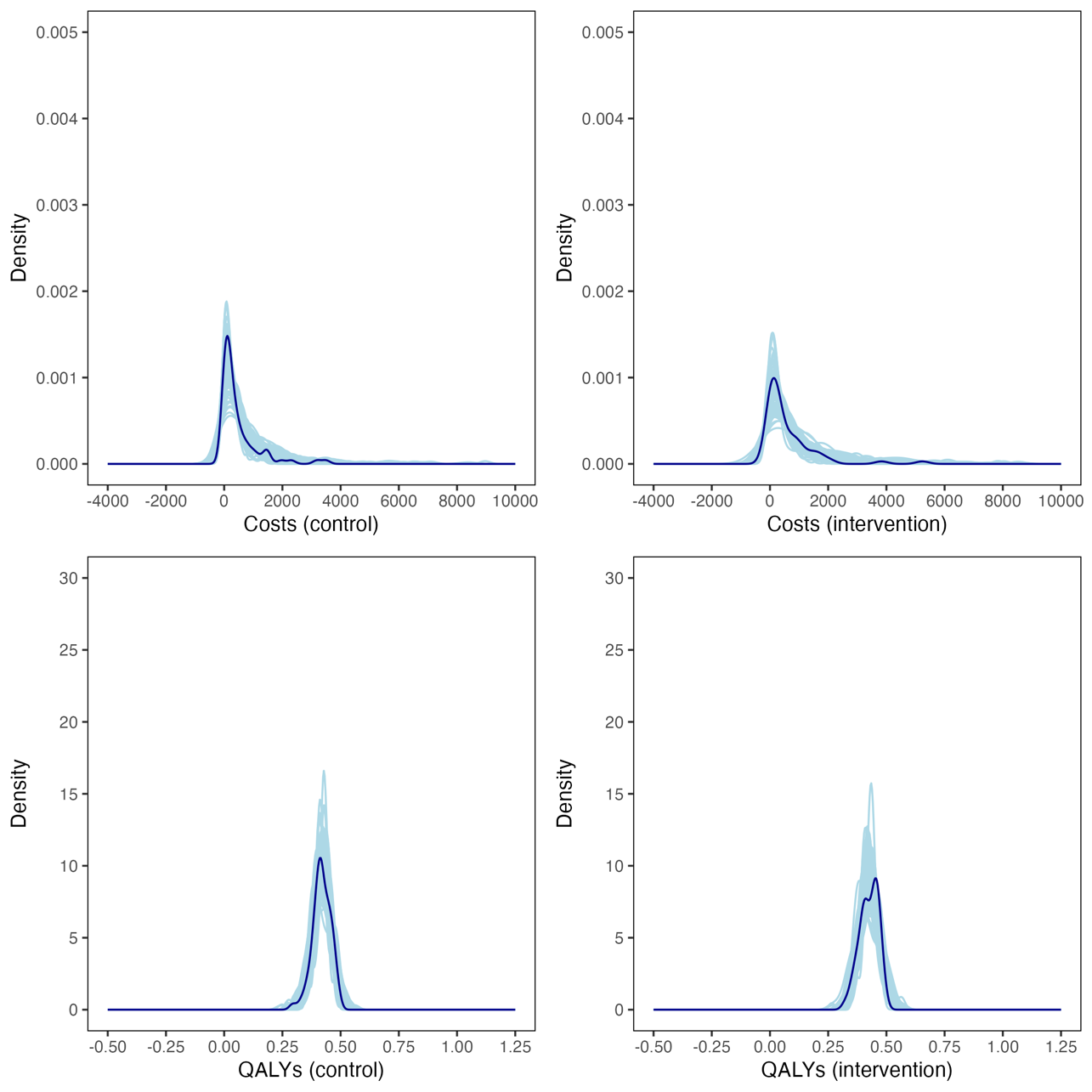


**Fig. 10** Distributions of replicated total health care costs and QALYs by treatment arm drawn from posterior predictive distribution compared to the distribution of observed data under the Beta Gamma model with Uniform(0,10000) as the prior on cost standard deviations. The dark blue curve represents observed data while the light blue curves display 100 simulated total health care costs and QALYs drawn from their posterior predictive distributions.


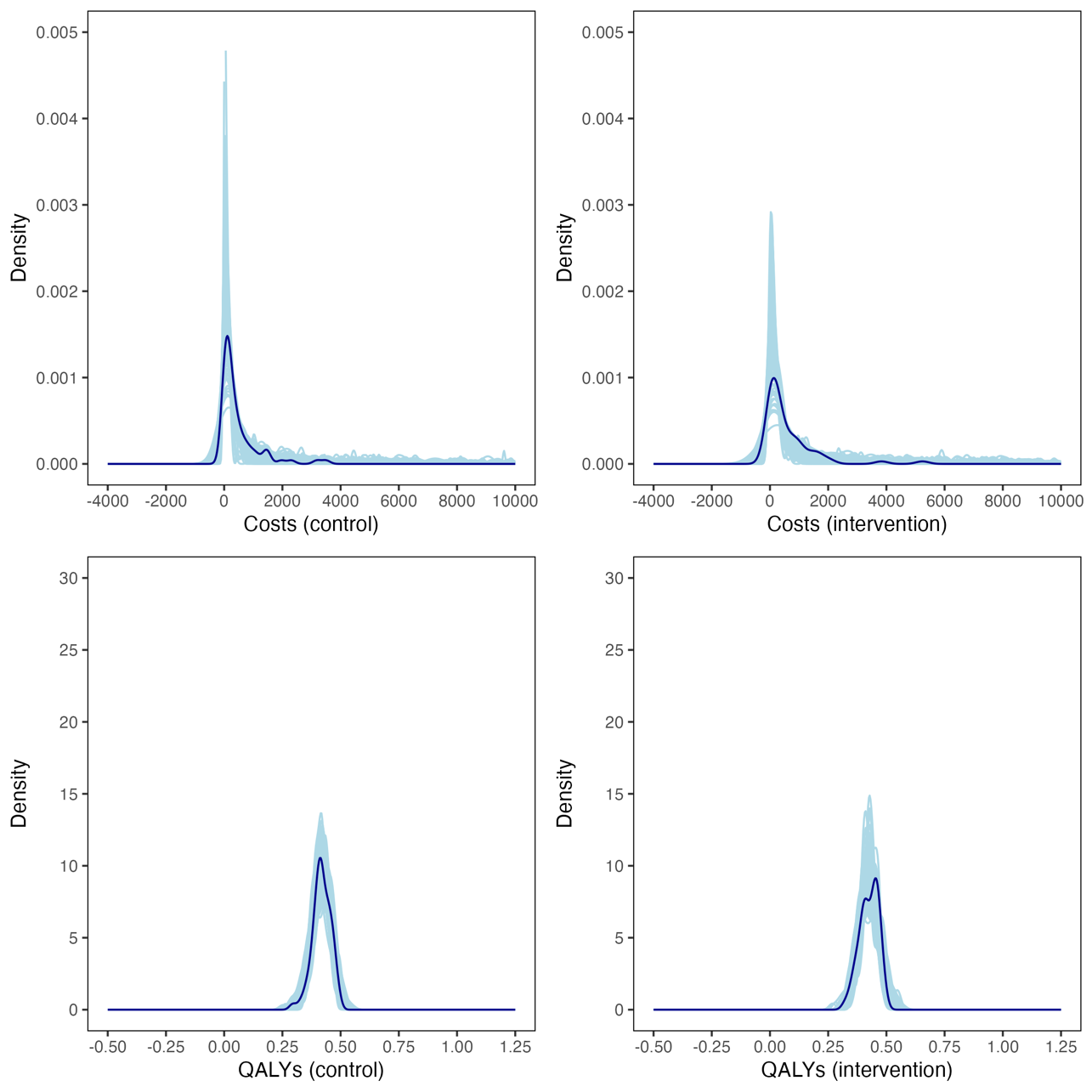


**Fig. 11** Distributions of replicated total health care costs and QALYs by treatment arm drawn from posterior predictive distribution compared to the distribution of observed data under the Beta Log-Normal model with Uniform(0,3) as the prior on log cost standard deviations. The dark blue curve represents observed data while the light blue curves display 100 simulated total health care costs and QALYs drawn from their posterior predictive distributions.


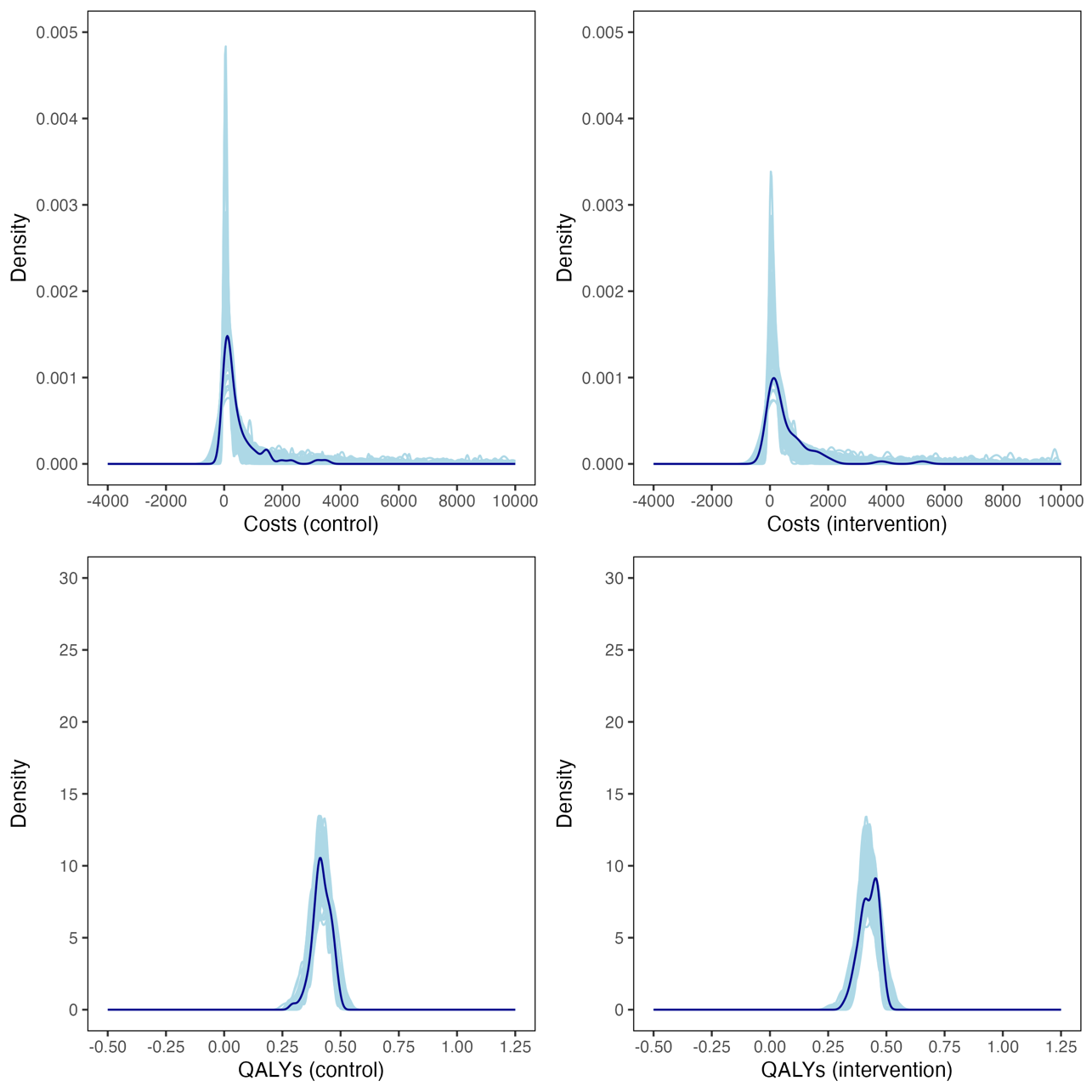


**Fig. 12** Distributions of replicated total health care costs and QALYs by treatment arm drawn from posterior predictive distribution compared to the distribution of observed data under the Beta Log-Normal model with Uniform(0,2) as the prior on log cost standard deviations. The dark blue curve represents observed data while the light blue curves display 100 simulated total health care costs and QALYs drawn from their posterior predictive distributions.


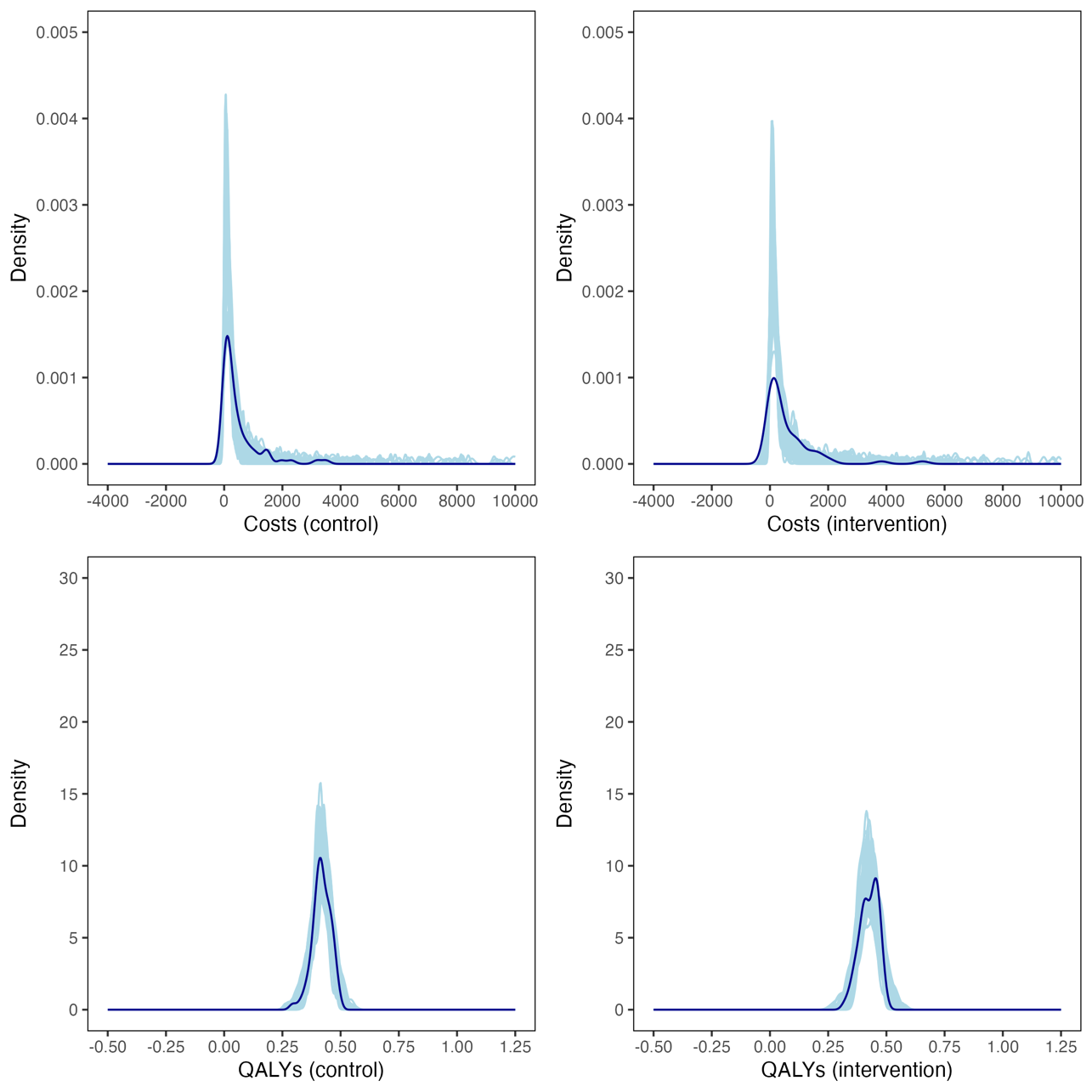


**Fig. 13** Distributions of replicated total health care costs and QALYs by treatment arm drawn from posterior predictive distribution compared to the distribution of observed data under the Beta Log-Normal model with Uniform(0,1) as the prior on log cost standard deviations. The dark blue curve represents observed data while the light blue curves display 100 simulated total health care costs and QALYs drawn from their posterior predictive distributions.


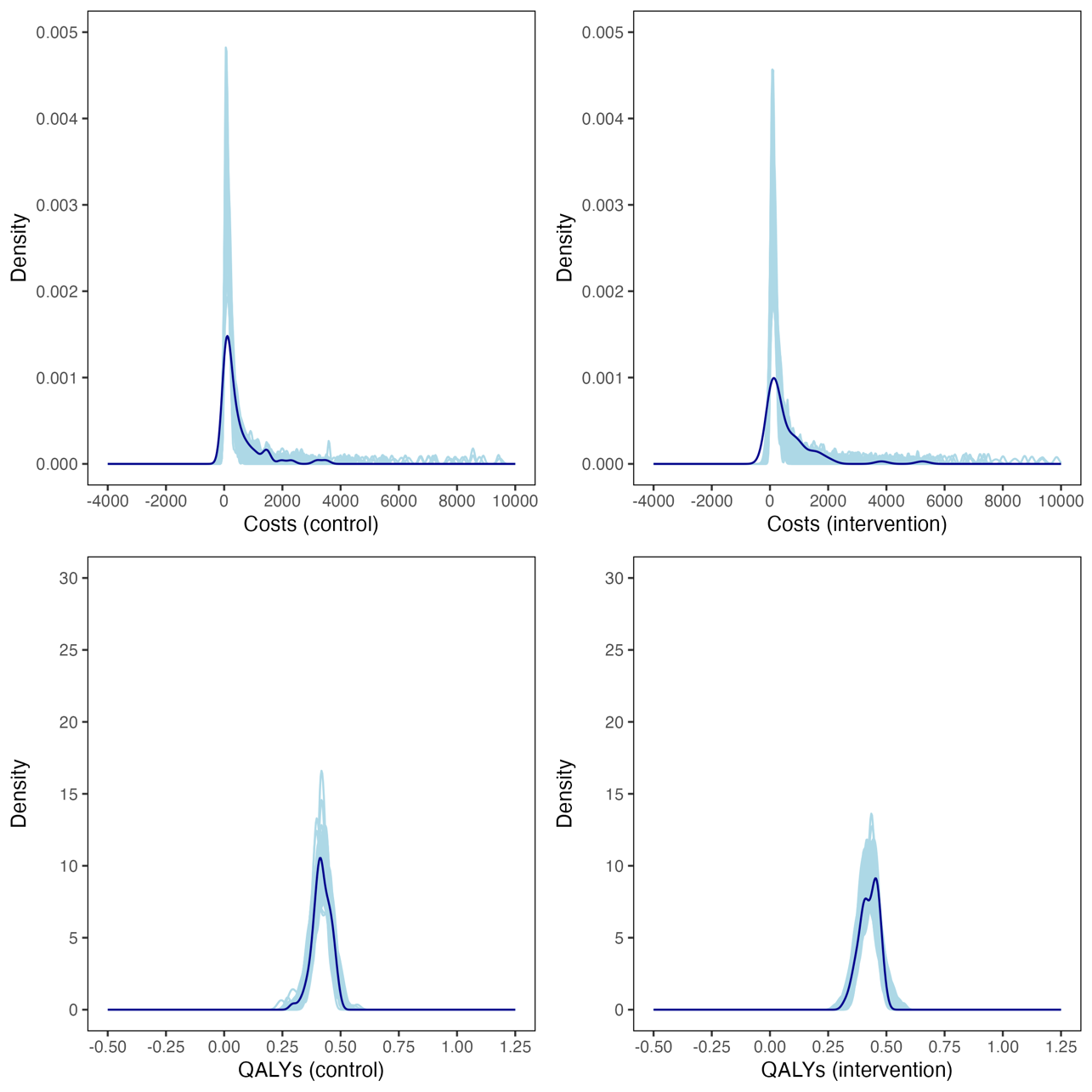


**Fig. 14** Distributions of replicated total health care costs and QALYs by treatment arm drawn from posterior predictive distribution compared to the distribution of observed data under the Beta Log-Normal model with Uniform(0,0.8) as the prior on log cost standard deviations. The dark blue curve represents observed data while the light blue curves display 100 simulated total health care costs and QALYs drawn from their posterior predictive distributions.

## Appendix D. Prior sensitivity analysis for model coefficients across alternative specifications

| Prior on Cost Standard Deviation | Prior on Coefficients in Cost Model | DIC | Costs (Con) | | Costs (Int) | | | Incremental Costs | |
| --- | --- | --- | --- | --- | --- | --- | --- | --- | --- |
| ***Normal model*** | | | | | | | | | |
| Uniform(0,1000) | Normal (${0,1000}^{2})$ | 947.6 | 482 | (333, 636) | 601 | (425, 786) | 119 | | (-108, 367) |
|  | Normal (${0,100}^{2})$ | 948.8 | 476 | (320, 632) | 598 | (421, 775) | 123 | | (-102, 366) |
|  | Normal (${0,10}^{2})$ | 959.9 | 367 | (214, 510) | 471 | (319, 636) | 104 | | (-110, 324) |
| Uniform(0,10000) | Normal (${0,1000}^{2})$ | 947.6 | 482 | (333, 636) | 601 | (425, 786) | 119 | | (-108, 367) |
|  | Normal (${0,100}^{2})$ | 948.8 | 476 | (320, 632) | 598 | (421, 775) | 123 | | (-102, 366) |
|  | Normal (${0,10}^{2})$ | 959.9 | 367 | (214, 510) | 471 | (319, 636) | 104 | | (-110, 324) |
| ***Gamma model*** | | | | | | | | | |
| Uniform(0,1000) | Normal (${0,100}^{2})$ | 718.7 | 537 | (388, 692) | 709 | (451, 970) | 171 | | (-128, 484) |
|  | Normal (${0,10}^{2})$ | 718.8 | 538 | (396, 703) | 711 | (468, 985) | 172 | | (-149, 477) |
| Uniform(0,10000) | Normal (${0,100}^{2})$ | 718.4 | 535 | (393, 697) | 710 | (469, 982) | 174 | | (-131, 480) |
|  | Normal (${0,10}^{2})$ | 718.3 | 537 | (387, 693) | 714 | (472, 993) | 177 | | (-129, 499) |
| ***Log-Normal model*** | | | | | | | | | |
| Uniform(0,3) | Normal (${0,100}^{2})$ | 744.4 | 2095 | (497, 4761) | 3396 | (620, 8279) | 1301 | | (-4169, 8699) |
|  | Normal (${0,10}^{2})$ | 744.3 | 1981 | (469, 4610) | 3243 | (630, 8116) | 1262 | | (-4511, 7754) |
| Uniform(0,2) | Normal (${0,100}^{2})$ | 740.1 | 1610 | (489, 3266) | 1954 | (651, 3868) | 344 | | (-2258, 3256) |
|  | Normal (${0,10}^{2})$ | 739.8 | 1537 | (477, 3076) | 1942 | (610, 3879) | 404 | | (-2223, 3185) |
| Uniform(0,1) | Normal (${0,100}^{2})$ | 919.3 | 412 | (260, 585) | 468 | (287, 667) | 56 | | (-208, 335) |
|  | Normal (${0,10}^{2})$ | 918.3 | 405 | (260, 585) | 468 | (287, 667) | 63 | | (-208, 335) |
| Uniform(0,0.8) | Normal (${0,100}^{2})$ | 1126.9 | 337 | (241, 453) | 386 | (265, 518) | 49 | | (-127, 215) |
|  | Normal (${0,10}^{2})$ | 1126.5 | 333 | (228, 436) | 387 | (270, 519) | 54 | | (-118, 224) |

**Table 4** Marginal mean and incremental mean cost estimates (and 95% credible intervals), for models with different Uniform prior distributions on cost standard deviations. Note: Costs are measured using British pound (£). DIC = Deviance Information Criteria; Con = Control; Int = Intervention.

## Appendix E. Details for the simulation study

The primary objective of this simulation study is to explore the sensitivity of the three most used cost model choices in cost-effectiveness analysis (i.e. the Normal, Gamma and Log-Normal model) to the priors on cost standard deviations in a health economics context.

1. Data Generating Process
   1. Set-Up

The simulation settings are carefully chosen to reflect common challenges in routine health economics evaluations. We consider a cost-effectiveness analysis alongside a one-site, six-month and two-arm RCT and build on previous simulation studies to generate individual-level cost-effectiveness data [1–3]. Individuals are randomly assigned to each treatment arm using a 1:1 allocation ratio.

For each subject, we assume the only continuous demographic variable, age, denoted as $age_{i}$, and baseline utilities, denoted as $u_{0i},$ to follow a bivariate Normal distribution:

$$\left( \begin{matrix} age_{i} \\ u_{0i} \end{matrix} \right)\sim N\left( \left( \begin{matrix} 12 \\ 0.82 \end{matrix} \right),\left( \begin{matrix} 2^{2} & -0.13\times2\times0.07 \\ -0.13\times2\times0.07 & {0.07}^{2} \end{matrix} \right) \right)$$

where $i$ is the individual indicator. Parameters have been calibrated to mimic the case study data and the theoretical properties of these covariates. For instance, the utility scores measured by the CHU-9D questionnaire range between 0.3261 and 1.000 in theory for a UK population[4]. Our data generating process leads to mean age at 12 years old and mean baseline utility scores at 0.817 per treatment arm when the number of participants is 200 while results in mean age at 12 years old while mean utilities at 0.820 per treatment arm when the sample size increases to 2000.

- 1. Simulation scenarios

Based on the three dimensions – i.e. data skewness, the proportion of zero and sample size – to explore in this simulation study, we have eight scenarios to explore. We set the marginal mean cost in control and intervention arm as £480 and £600 respectively, resulting in an incremental cost at £120. The cost standard deviations have been set to British pounds 700 and 900 for the control and intervention group, respectively.

First, we generate QALYs from a Normal distribution, with $\mu_{ei}$ and $\sigma_{e}$ representing the individual-specific mean and population-specific standard deviation, respectively. The data generating process can be specified as:

$$\begin{matrix} e_{i} & \sim\text{Normal}\left( \mu_{ei},\sigma_{e}^{2} \right) \\ \mu_{ei} & =0.4+0.05trt_{i}-0.007age_{i}+0.{26u}_{0i} \end{matrix}$$

where $\sigma_{e}=0.2$ and $trt_{i}$ is the individual-specific treatment indicator. The resulting mean QALYs are 0.404 and 0.446 for the control and intervention arm, respectively, with a small sample size at 200 and become 0.401 and 0.452, respectively, when the sample size is 2000.

Second, the proportion of zero values are varied across scenarios, leading to different cost data. In cases where the proportion of zero values is 10%, we model the probability of having zero cost for individuals using Bernoulli distributions with a logit link function:

$$\begin{matrix} p_{i} & \sim\text{Bernoulli}\left( \pi_{i} \right) \\ \mathrm{logit}\left( \pi_{i} \right) & =\gamma_{0}+\gamma_{1}trt_{i}+\gamma_{2}age_{i}+\gamma_{3}u_{0i} \end{matrix}$$

where $p_{i}$ is the indicator of whether cost for an individual is zero or not, $\pi_{i}$ denotes the probability of cost being zero, $\boldsymbol{\gamma}=(\gamma_{0}, \gamma_{1}, \gamma_{2}, \gamma_{3})$ is the set of intercept and coefficient parameters in the logistic regression. We calibrate the values of the $\boldsymbol{\gamma}$ so that the generated cost data can match the desired proportions of zeros (Table 5).

| **Proportion of zero values in cost data** | N=200 | N=2000 |
| --- | --- | --- |
| 10% | $\gamma_{0}=0.19,\gamma_{1}=-0.21, \gamma_{2}=-0.20,\gamma_{3}=0.15$ | $\gamma_{0}=0.06,\gamma_{1}=0.11, \gamma_{2}=-0.20,\gamma_{3}=0.06$ |

**Table 5** Parameter values for the logistic regression to generate zeros across scenarios containing zero cost values.

The positive component of the cost data is generated either from Log-Normal or Gamma distributions, and re-parametrised based on an individual-level mean parameter ($\mu_{ci}$) and a population-level standard deviation ($\sigma_{c}$). When the cost data follow a Gamma distribution, a log link is used. The exact model specification is the same as the cost components of the Beta Gamma and Beta Log-Normal models described in the case study. A summary of the data generating process can be written as:

$$\begin{matrix} c_{i}\mid e_{i} & \sim\text{dist}\left( \mu_{ci},\sigma_{c} \right) \\ g\left( \mu_{ci} \right) & =\beta_{0}+\beta_{1}{trt}_{i}+\beta_{2}{age}_{i}+\beta_{3}e_{i} \end{matrix}$$

where $\boldsymbol{\beta}=(\beta_{0}, \beta_{1},\beta_{2},\beta_{3})$ is the set of parameters that indexes the regression equation of the cost models. The $\boldsymbol{\beta}$ has been calibrated to ensure the true parameter values align with the study design (Table 6). The Log-Normal and Gamma models will also be directly applied to scenarios where there is no zero value in the cost data.

| **Cost distributions and proportion of zeros** | **Parameter values** | |
| --- | --- | --- |
|  | N=200 | N=2000 |
| *Log-Normal distribution* | | |
| 0% | $\beta_{0}=5.60,\beta_{1}=0.204, \beta_{2}=-0.02,\beta_{3}=-0.68$ | $\beta_{0}=5.60,\beta_{1}=0.204, \beta_{2}=-0.02,\beta_{3}=-0.72$ |
| 10% | $\beta_{0}=5.78,\beta_{1}=0.205, \beta_{2}=-0.11,\beta_{3}=-3.94$ | $\beta_{0}=5.80,\beta_{1}=0.205, \beta_{2}=-0.11,\beta_{3}=-3.94$ |
| *Gamma distribution* |  |  |
| 0% | $\beta_{0}=6.08,\beta_{1}=0.2230, \beta_{2}=0.16,\beta_{3}=-2.02$ | $\beta_{0}=6.08,\beta_{1}=0.2230, \beta_{2}=0.16,\beta_{3}=-2.02$ |
| 10% | $\beta_{0}=6.03,\beta_{1}=0.2224, \beta_{2}=0.27,\beta_{3}=-2.94$ | $\beta_{0}=6.03,\beta_{1}=0.2224, \beta_{2}=0.27,\beta_{3}=-2.94$ |

**Table 6** Parameter values for the cost data generating process.

1. Methods

The Normal, Gamma, and Log-Normal models using different Uniform prior distributions on cost standard deviations will be performed and compared across scenarios. Specifically, we consider eight models: the Normal models with Uniform(0,1000) and Uniform(0,10000), the Gamma models with Uniform(0,1000) and Uniform(0,10000), and the Log-Normal models with Uniform(0,3), Uniform(0,2), Uniform(0,1) and Uniform(0,0.8). A constant of one will be added to the original data if the simulated dataset contains zero values.

These models are fitted in JAGS with the following MCMC parameter specifications: two chains, with the number of iterations, burn-in, and thinning rate chosen based on sample size. For larger samples (N = 2000), each chain runs for 7000 iterations, with a burn-in of 2000 and a thinning rate of 5, resulting in 2000 iterations for inference. For smaller samples (N = 200), more iterations are required: we use 20,000 iterations per chain, a burn-in of 10,000, and a thinning rate of 2, yielding 10,000 iterations for inference.

1. Performance Measures

The performance of different statistical methods is assessed by bias, empirical standard errors, and root mean squared error (RMSE). These performance measures are defined as below [5]:

|  | **Estimate** |
| --- | --- |
| **Bias** | $\frac{1}{n_{sims}}\sum_{i=1}^{n_{sims}} \hat{\theta}_{i}-\theta$ |
| **Empirical standard error (EmpSE)** | $\sqrt{\frac{1}{n_{sims}-1}\sum_{i=1}^{n_{sim}} \left( \hat{\theta}_{i}-\bar{\theta} \right)^{2}}$ |
| **Root mean squared error (RMSE)** | $\sqrt{\frac{1}{n_{sims}}\sum_{i=1}^{n_{sim}} \left( \hat{\theta}_{i}-\theta\right)^{2}}$ |

**Table 7** Definitions of performance measures. $n_{sims}$ represents the number of simulations. $\theta$ is the true parameter value, $\hat{\theta}_{i}$ is the estimate of $\theta$ from $i$th simulation, and $\bar{\theta}$ is the mean of $\hat{\theta}_{i}$ across $n_{sims}$ simulations.

1. Results

| **Models** | **Scenarios** | | | | | | | |
| --- | --- | --- | --- | --- | --- | --- | --- | --- |
|  | **10% Zeros,**  **N = 200** | | **No Zero,**  **N = 200** | | **10% Zeros,**  **N = 2000** | | **No Zero,**  **N = 2000** | |
|  | **Empirical SE** | **RMSE** | **Empirical SE** | **RMSE** | **Empirical SE** | **RMSE** | **Empirical SE** | **RMSE** |
| ***Normal model*** | | | | | | | | |
| Uniform(0,1000) | 40 | 133 | 42 | 124 | 28 | 119 | 29 | 86 |
| Uniform(0,10000) | 48 | 148 | 45 | 142 | 27 | 120 | 28 | 87 |
| ***Gamma model*** | | | | | | | | |
| Uniform(0,1000) | 67 | 113 | 79 | 112 | 27 | 123 | 30 | 72 |
| Uniform(0,10000) | 111 | 125 | 115 | 120 | 34 | 115 | 33 | 70 |
| ***Log-Normal model*** | | | | | | | | |
| Uniform(0,3) | 1692 | 1753 | 11,238,106 | 11,274,966 | 457 | 480 | 48,404 | 55,437 |
| Uniform(0,2) | 371 | 489 | 768,007 | 770,666 | 155 | 694 | 3,576 | 3,805 |
| Uniform(0,1) | 85 | 191 | 157,028 | 157,564 | 35 | 253 | NA | NA |
| Uniform(0,0.8) | 71 | 177 | 130,043 | 130,486 | 29 | 231 | NA | NA |

**Table 8** Relative performance in incremental costs of Normal, Gamma, and Log-Normal models with different Uniform prior distributions when cost data follow a Gamma distribution. Empirical SE = Empirical Standard Error; RMSE = Root Mean Squared Error.

| **Models** | **Scenarios** | | | | | | | | | | | |
| --- | --- | --- | --- | --- | --- | --- | --- | --- | --- | --- | --- | --- |
|  | **10% Zeros, N = 200** | | | **No Zero, N = 200** | | | **10% Zeros, N = 2000** | | | **No Zero, N = 2000** | | |
|  | **Bias** | **Empirical SE** | **RMSE** | **Bias** | **Empirical SE** | **RMSE** | **Bias** | **Empirical SE** | **RMSE** | **Bias** | **Empirical SE** | **RMSE** |
| ***Normal model*** | | | | | | | | | | | | |
| Uniform(0,1000) | -167 | 170 | 27 | -165 | 28 | 167 | -1 | 18 | 18 | -9 | 19 | 21 |
| Uniform(0,10000) | -170 | 173 | 31 | -168 | 30 | 170 | -1 | 18 | 18 | -9 | 19 | 21 |
| ***Gamma model*** | | | | | | | | | | | | |
| Uniform(0,1000) | 18 | 52 | 49 | 6 | 59 | 59 | 82 | 21 | 85 | 16 | 21 | 26 |
| Uniform(0,10000) | 40 | 81 | 70 | 17 | 71 | 73 | 82 | 21 | 85 | 16 | 21 | 26 |
| ***Log-Normal model*** | | | | | | | | | | | | |
| Uniform(0,3) | 2,896 | 3,076 | 1,037 | 50,155 | 108,294 | 119,296 | 2,924 | 299 | 2940 | 48,249 | 17,577 | 51,347 |
| Uniform(0,2) | 870 | 913 | 276 | 3,615 | 7,926 | 8,709 | 1,351 | 122 | 1356 | 3,730 | 1,512 | 4,025 |
| Uniform(0,1) | -168 | 180 | 64 | 374 | 1,594 | 1,636 | -67 | 28 | 73 | NA | NA | NA |
| Uniform(0,0.8) | -221 | 227 | 53 | 227 | 1,316 | 1,334 | -136 | 23 | 138 | NA | NA | NA |

**Table 9** Relative performance in mean costs (control) of Normal, Gamma, and Log-Normal models with different Uniform prior distributions when cost data follow a Gamma distribution. Empirical SE = Empirical Standard Error; RMSE = Root Mean Squared Error.

| **Models** | **Scenarios** | | | | | | | | | | | |
| --- | --- | --- | --- | --- | --- | --- | --- | --- | --- | --- | --- | --- |
|  | **10% Zeros, N = 200** | | | **No Zero, N = 200** | | | **10% Zeros, N = 2000** | | | **No Zero, N = 2000** | | |
|  | **Bias** | **Empirical SE** | **RMSE** | **Bias** | **Empirical SE** | **RMSE** | **Bias** | **Empirical SE** | **RMSE** | **Bias** | **Empirical SE** | **RMSE** |
| ***Normal model*** | | | | | | | | | | | | |
| Uniform(0,1000) | -294 | 296 | 29 | -282 | 31 | 283 | -116 | 26 | 119 | -90 | 22 | 93 |
| Uniform(0,10000) | -311 | 313 | 37 | -302 | 34 | 304 | -117 | 21 | 119 | -91 | 21 | 93 |
| ***Gamma model*** | | | | | | | | | | | | |
| Uniform(0,1000) | -73 | 86 | 45 | -74 | 50 | 89 | -38 | 18 | 42 | -50 | 22 | 55 |
| Uniform(0,10000) | -16 | 87 | 86 | -16 | 89 | 90 | -28 | 27 | 38 | -46 | 26 | 53 |
| ***Log-Normal model*** | | | | | | | | | | | | |
| Uniform(0,3) | 3,355 | 3,615 | 1,345 | 1,027,976 | 11,235,032 | 11,276,367 | 2,779 | 357 | 2,801 | 75,318 | 44,845 | 87,646 |
| Uniform(0,2) | 550 | 602 | 244 | 72,023 | 768,008 | 770,767 | 674 | 99 | 682 | 5,036 | 3,220 | 5,977 |
| Uniform(0,1) | -340 | 344 | 56 | 14,277 | 157,028 | 157,551 | -318 | 22 | 319 | NA | NA | NA |
| Uniform(0,0.8) | -384 | 386 | 46 | 11,724 | 130,043 | 130,468 | -365 | 18 | 365 | NA | NA | NA |

**Table 10** Relative performance in mean costs (intervention) of Normal, Gamma, and Log-Normal models with different Uniform prior distributions when cost data follow a Gamma distribution. Empirical SE = Empirical Standard Error; RMSE = Root Mean Squared Error.

[1] Gomes M, Ng E, Grieve R, Nixon R, Carpenter J, Thompson S. Developing Appropriate Methods for Cost-Effectiveness Analysis of Cluster Randomized Trials. Med Decis Making 2012;32:350–61. https://doi.org/10.1177/0272989X11418372.

[2] Kreif N, Grieve R, Radice R, Sekhon J. Regression-adjusted matching and double-robust methods for estimating average treatment effects in health economic evaluation. Health Serv Outcomes Res Methodol 2013;13:174–202. https://doi.org/10.1007/s10742-013-0109-2.

[3] Gabrio A, Hunter R, Mason A, Baio G. Joint Longitudinal Models for Dealing With Missing at Random Data in Trial-Based Economic Evaluations. Value Health 2021;24:699–706. https://doi.org/10.1016/j.jval.2020.11.018.

[4] Petersen KD, Ratcliffe J, Chen G, Serles D, Frøsig CS, Olesen AV. The construct validity of the Child Health Utility 9D-DK instrument. Health Qual Life Outcomes 2019;17:187. https://doi.org/10.1186/s12955-019-1256-0.

[5] Morris T, White I, Crowther M. Using simulation studies to evaluate statistical methods. Stat Med 2019;38:29. https://doi.org/10.1002/sim.8086.
